# Evaluation of SI, MSI and DSI for very early (3-day) mortality in patients with septic shock

**DOI:** 10.1186/s40001-022-00857-y

**Published:** 2022-11-03

**Authors:** Tie-Ning Zhang, Peng-Hui Hao, Shan-Yan Gao, Chun-Feng Liu, Ni Yang

**Affiliations:** 1 Department of Pediatrics, Shengjing Hospital of China Medical University, No. 36, San Hao Street, Shenyang, 110004 Liaoning People’s Republic of China; 2 Department of Clinical Epidemiology, Shengjing Hospital of China Medical University, Shenyang, China; 3 Clinical Research Center, Shengjing Hospital of China Medical University, Shenyang, China

**Keywords:** Diastolic shock index, Modified shock index, Mortality, Septic shock, Shock index

## Abstract

**Background:**

Septic shock is associated with increased mortality. Predicting mortality, including early prediction for septic shock patients in intensive care units (ICUs), remains an important challenge.

**Method:**

We searched the Medical Information Mart for Intensive Care IV database. Odds ratios (ORs) with 95% confidence intervals (CIs) of the relationships between shock index (SI), modified SI (MSI), and diastolic SI (DSI) of patients with septic shock requiring vasopressors and 3-day/in-hospital mortality were calculated using logistic regression models. The time-course changes of these parameters were compared between survivors and non-survivors. The performance of the different parameters was described by the area under the receiver operating characteristic (ROC) curve (AUC) and compared with DeLong analysis.

**Results:**

A total of 1266 patients with septic shock requiring vasopressors were identified. The 3-day mortality rate and in-hospital mortality rate were 8.7% and 23.5%, respectively. Multivariable logistic regression analysis showed significant associations between pre-vasopressor SI/MSI/DSI and 3-day mortality in patients with septic shock requiring vasopressors in fully adjusted models (*P*s for trend < 0.01). The AUCs of pre-vasopressor SI, MSI, and DSI were 0.746, 0.710, and 0.732 for 3-day mortality, respectively. There were significant differences in the time-course of SI, MSI, and DSI between survivors and non-survivors at 3-day/in-hospital mortality among patients with septic shock requiring vasopressors (repeated-measures ANOVA, inter-subjects difference *P* < 0.001).

**Conclusion:**

Pre-vasopressor SI, MSI, and DSI values identified patients with septic shock requiring vasopressors who are at increased risk of early death. Of these easy-to-acquire values, SI and MSI show a comparatively better performance.

**Supplementary Information:**

The online version contains supplementary material available at 10.1186/s40001-022-00857-y.

## Background

Sepsis is defined as life-threatening organ dysfunction caused by a dysregulated host response to infection, which is common in the intensive care unit (ICU) [1]. Septic shock is a subset of sepsis in which the underlying circulatory and cellular abnormalities are profound enough to greatly increase mortality [1]. Notably, despite the development and improvement of clinical equipment and technology, as well as the advance of medical care, the incidence and mortality due to sepsis, especially septic shock, remain high. According to the latest international guidelines for the management of sepsis and septic shock published in 2021, early identification and appropriate management in the initial hours after the development of sepsis improves outcomes [2]. Therefore, identifying patients with septic shock who are at increased risk of early death can direct the priority of care, assisting those who are likely to benefit from higher levels of care. Despite the large number of studies focusing on biomarkers and clinical prediction tools for predicting in-hospital mortality of sepsis patients [3–5], the early identification of septic shock patients at increased risk remains challenging. Therefore, studies exploring non-invasive hemodynamic parameters or tools that can be easily used in the clinical settings, and validating their performance, are urgently needed.

Previous studies have suggested that persistently low mean arterial pressure (MAP), systolic blood pressure (SBP), and diastolic blood pressure (DBP) are associated with worse prognosis in patients with septic shock [6–9]. Recently, the study performed by Ospina-Tascón et al. reported that a novel index, the “diastolic shock index (DSI)”, defined as the ratio of heart rate (HR) and DBP, was related to 90-day mortality in patients with septic shock [10]. However, whether the DSI performs well in predicting very early mortality (for example, 3-day mortality) remains unclear. In addition, the shock index (SI) defined as the ratio of HR to SBP, and the modified shock index (MSI) obtained by dividing HR by MAP, have been studied in patients at risk of or experiencing shock from various causes, including sepsis [11]. Related studies have shown that the SI and MSI may have their own roles in identifying patients in the emergency department (ED) who are at increased risk of mortality [12, 13]. Of note, whether pre-VPs SI, MSI, and DSI values could identify patients with septic shock requiring vasopressors who are at increased risk of early death is unknown. Considering that SI, MSI, and DSI can be easily obtained in clinical practice, their potential value in predicting the prognosis of patients with septic shock very early may be useful to identify patients early on at high risk for mortality in order to ensure prompt admission/transfer to an appropriate level of care, and also help inform conversations about prognosis with patients and their families.

Therefore, this study aimed to evaluate the relationships between non-invasive hemodynamic parameters and 3-day/in-hospital mortality for patients with septic shock, hypothesizing that pre-VPs SI, MSI, and DSI values could identify patients with septic shock requiring vasopressors who are at increased risk of early death.

## Materials and methods

### Study population

This was a restrictive observational study using data from the Medical Information Mart for Intensive Care IV (MIMIC-IV version 1.0) database from 2008 to 2019 [14]. MIMIC-IV, an update to the MIMIC-III, is a real-world publicly available clinical database maintained by Beth Israel Deaconess Medical Center, listing more than 60,000 ICU incidents. The database was accessed by an individual who has completed the Collaborative Institutional Training Initiative examination (Certification number 39022265 for Hao). All data were extracted using the SQL programming language. This longitudinal, single-center database included 76,540 patients who were admitted to an ICU. We included only patients admitted to the ICU for the first time. All intensive care patients diagnosed with septic shock were screened and identified by the “long_title” in the “d_icd_diagnoses” table of MIMIC-IV database. Our study followed the Strengthening the Reporting of Observational Studies in Epidemiology (STROBE) guidelines [15]. The code used for data extraction is available at GitHub (https://github.com/MIT-LCP/mimic-iv).

The inclusion criteria of our study were: adult patients (> 18 years old) with septic shock and treatment with vasopressors. The exclusion criteria were: (1) age less than 18 years; (2) diagnosis of malignant arrhythmia, which can lead to cardiogenic shock; (3) history of ischemic heart disease; (4) diagnosis of cardiomyopathy; (5) hepatic-related diseases such as liver cirrhosis; (6) history of chronic renal disease; (7) shock other than septic shock, and (8) pregnancy.

### Data extraction

We extracted patient parameters, including age, sex, weight, race, type of ICU, the first 24 h Sequential Organ Failure Assessment (SOFA) score, the Oxford Acute Severity of Illness Score (OASIS), the Logistic Organ Dysfunction Score (LODS), the Charlson comorbidity index, interventions [i.e., renal replacement therapy (RRT), mechanical ventilation use, and VPs use], vital signs (i.e., MAP, SBP, DBP, HR, and temperature), initial lactate level, initial arterial pH, net fluid balance, fluid input at day 1, and urine output. Notably, we also extracted vital signs including MAP, SBP, DBP, and HR at 1 h, 2 h, 4 h, 8 h, 12 h, 24 h, 48 h, and 72 h after treatment initiation with VPs.

### Study covariates and outcomes

The SI was calculated as the quotient between HR and SBP. MSI was calculated as the ratio between HR and MAP. DSI was calculated as the ratio between HR and DBP. These three parameters were calculated before VP treatment, and at 1 h, 2 h, 4 h, 8 h, 12 h, 24 h, 48 h, and 72 h after VP treatment. The patients could receive VP treatment before or after admission to the ICU. The parameters of pre-VP treatment were calculated as the average values of the patient data that were within 6 h before admission to the ICU until VP treatment. The primary endpoint of interest was the 3-day mortality rate of patients with septic shock requiring vasopressors. The secondary endpoint was in-hospital mortality.

### Statistical analysis

Continuous variables were described using the least-squares mean and 95% confidence intervals (CIs). Categorical variables are presented as percentages. Logistic regression analyses were used to compare patient characteristics and outcomes according to the quartiles of pre-VP SI, DSI, and MSI. Odds ratios (ORs) with 95% CIs of the relationships between pre-VP SI, DSI, and MSI and 3-day/in-hospital mortality rates were calculated using logistic regression models. When pre-VP SI, DSI, and MSI were modeled as a quartile-based categorical variable, the lowest quartile was set as the reference group in each model. The significance of the linear trends of 3-day/in-hospital mortality rate across categories of pre-VP SI, DSI, and MSI was examined by assigning the median value to each quartile, and these variables were analyzed as a continuous variable in multivariate models. Multivariable logistic models were prepared as follows: Model 1 was adjusted for age, sex, race, and type of ICU care unit. Model 2 was further adjusted for the SOFA score on day-1 based on Model 1. Repeated-measures ANOVA was used to evaluate time-course differences in SI, MSI, and DSI pre-VPs and at 1 h, 2 h, 4 h, 8 h, 12 h, 24 h, 48 h, and 72 h after VP treatment between 3-day/in-hospital septic shock requiring vasopressors survivors and non-survivors. The performance of SI, MSI, and DSI pre-VPs, and at 1 h, 2 h, 4 h, 8 h, and 12 h after VP treatment were described by the area under the receiver operating characteristic (ROC) curve (AUC) and compared with DeLong analysis. Youden’s index was used to determine optimal cut-off values. Statistical significance was set at *P* < 0.05. All analyses were conducted using the SAS version 9.4 software (SAS Institute, Cary, NC, USA).

## Results

### Study population

**Fig. 1:**
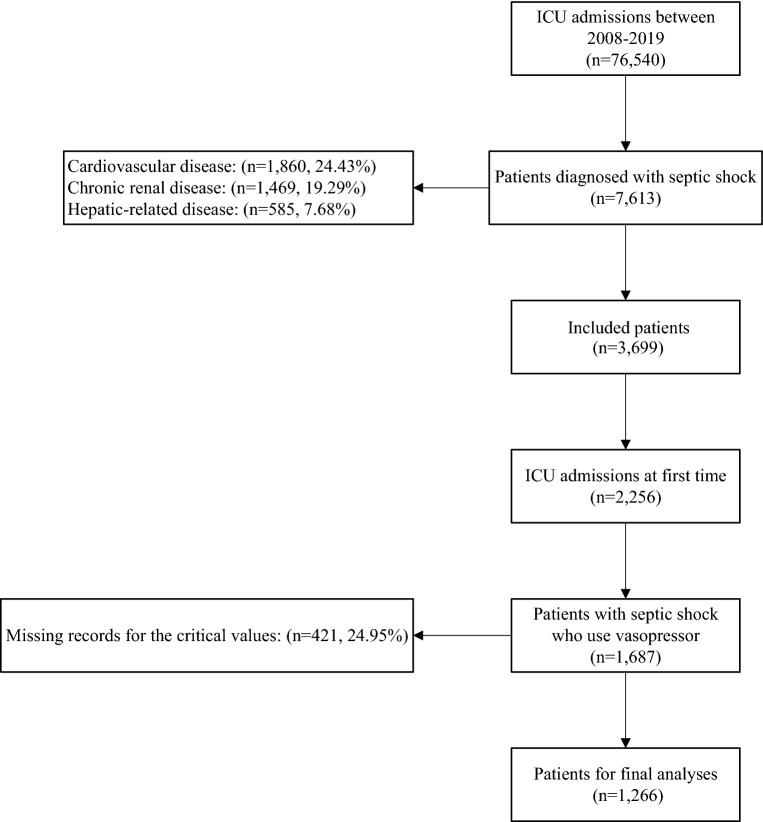
Flowchart of selection of patients included in this study

After reviewing 76,540 MIMIC-IV database ICU admissions, we identified 7,613 patients diagnosed with septic shock. After including only patients’ first ICU admissions with septic shock requiring vasopressors and excluding missing records for the critical values, we finally included 1266 patients in our study (Fig. 1). Of these, 632 (49.9%) were male, and their mean (95% CI) of age was 66 (65 to 67) years. The means (95% CIs) of hospital and ICU length of stay were 15 (14 to 16) and 7 (7 to 8) days, respectively. The 3-day mortality rate and in-hospital mortality rate were 8.7% and 23.5%, respectively.

Table 1 summarizes the characteristics of patients with septic shock requiring vasopressors in the current MIMIC-IV database by quartiles of SI before the start of VPs (pre-VP SI), including several parameters that could reflect microcirculatory perfusion state in septic shock such as initial arterial pH, net fluid balance, fluid input and urine output. Individuals with higher pre-VP SI tended to use RRT and VPs at day 1, and had a lower age, initial arterial pH, urine output, pre-VPs MAP, SBP, and DBP; they also had a higher 3-day/in-hospital mortality, SOFA score (at day 1), OASIS, LODS, initial lactate level, net fluid balance (at day 1), norepinephrine max dose, fluid input (at day 1), pre-VP HR, and temperature (all *P* values < 0.05).

**Table 1:** Characteristics of 1,266 septic shock patients by quartiles of shock index before the start of vasopressor therapy

Covariate	Overall	Quartiles of pre-VPs SI				*P* for trend ^a^
		Q1	Q2	Q3	Q4	
*n*	1266	316	317	315	318	
Pre-VPs SI	0.96 (0.94, 0.97) ^b^	0.64 (0.63, 0.65)	0.84 (0.84, 0.85)	1.02 (1.01, 1.02)	1.32 (0.30, 1.34)	
Patient outcomes						
3-day mortality, n (%)	110 (8.7)	9 (0.7)	29 (2.3)	22 (1.7)	50 (4.0)	< 0.0001
Hospital mortality, *n* (%)	297 (23.5)	60 (4.7)	73 (5.8)	72 (5.7)	92 (7.3)	0.03
Hospital LOS (days)	15.11 (14.1, 16.11)	14.89 (12.26, 17.52)	12.98 (11.51, 14.45)	16.42 (14.46, 18.38)	16.14 (14.29, 17.99)	0.07
ICU LOS (days)	7.42 (6.91, 7.93)	8.07 (6.98, 9.16)	6.99 (5.87, 8.10)	6.92 (6.11, 7.72)	7.70 (6.65, 8.75)	0.32
Mechanical ventilation-free days	4.14 (3.82, 4.45)	4.47 (0.81, 5.12)	3.97 (3.24, 4.71)	4.01 (3.55, 4.47)	4.09 (3.48, 4.71)	0.68
RRT-free days	6.19 (5.79, 6.59)	6.59 (5.65, 7.54)	5.83 (5.07, 6.59)	6.04 (5.38, 6.71)	6.30 (5.45, 7.14)	0.59
Patient characteristics						
Age (years)	66.05 (65.16, 66.93)	69.92 (68.25, 71.58)	67.33 (65.60, 69.07)	63.69 (61.82, 65.56)	63.25 (61.52, 64.99)	< 0.0001
Male, *n* (%)	632 (49.9)	166 (13.1)	154 (12.2)	152 (12.0)	160 (12.6)	0.69
Weight (kg)	83.24 (81.84, 84.65)	83.09 (80.01, 86.17)	82.93 (80.15, 85.71)	83.77 (81.01, 86.53)	83.19 (80.52, 85.86)	0.98
Race, *n* (%)						0.77
White	820 (64.8)	204 (16.1)	208 (16.4)	203 (16.0)	205 (16.2)	
Asian	50 (4.0)	7 (0.6)	12 (1.0)	14 (1.1)	17 (1.3)	
Black/African American	109 (8.6)	29 (2.3)	23 (1.8)	27 (2.1)	30 (2.4)	
Hispanic/Latino	37 (2.9)	6 (0.5)	6 (0.5)	12 (1.0)	13 (1.0)	
American Indian/Alaska native	4 (0.3)	3 (0.2)	1 (0.1)	0	0	
Other	246 (19.4)	67 (5.3)	67 (5.3)	59 (4.7)	53 (4.2)	
SOFA score day-1	8.16 (7.95, 8.37)	7.10 (6.71, 7.49)	7.80 (7.38, 8.21)	8.41 (7.97, 8.85)	9.39 (8.97, 9.81)	< 0.0001
OASIS score	40.43 (39.85, 41.00)	37.65 (36.58, 38.72)	38.64 (37.50, 39.79)	40.67 (39.52, 41.82)	44.73 (43.62, 45.84)	< 0.0001
LODS score	8.17 (7.95, 8.40)	7.28 (6.86, 7.71)	7.75 (7.32, 8.19)	8.14 (7.70, 8.58)	9.51 (9.05, 9.97)	< 0.0001
Charlson comorbidity index	5.22 (5.07, 5.37)	5.48 (5.20, 5.76)	5.17 (4.88, 5.45)	5.01 (4.70, 5.32)	5.21 (4.90, 5.52)	0.17
Interventions, *n* (%)						
RRT use (1st 24 h)	81 (6.4)	10 (0.8)	15 (1.2)	23 (1.8)	33 (2.6)	< 0.01
Mechanical ventilation use (1st 24 h)	612 (48.3)	138 (10.9)	155 (12.2)	152 (12.0)	167 (13.2)	0.17
Vasopressor use (1st 24 h)	1107 (87.4)	263 (20.7)	269 (21.3)	277 (21.9)	298 (23.5)	< 0.001
Pre-VPs vital signs						
MAP (mmHg)	99 (97.86, 100.14)	82.70 (80.84, 84.55)	74.94 (73.45, 76.44)	70.85 (69.49, 72.22)	64.76 (63.45, 66.06)	< 0.0001
SBP (mmHg)	107.13 (106.08, 108.17)	123.58 (121.51, 125.65)	110.61 (108.9, 112.31)	102.18 (100.77, 103.59)	92.22 (90.82, 93.62)	< 0.0001
DBP (mmHg)	61.18 (60.35, 62.00)	67.74 (65.78, 69.70)	61.67 (60.09, 63.25)	59.88 (58.44, 61.32)	55.45 (54.15, 56.75)	< 0.0001
HR (bpm)	73.3 (72.46, 74.14)	78.55 (77.02, 80.07)	93.36 (91.86, 94.87)	103.87 (102.43, 105.31)	120.12 (118.43, 121.80)	< 0.0001
Temperature (°C)	36.92 (36.86, 36.98)	36.70 (36.57, 36.82)	36.85 (36.74, 36.97)	36.95 (36.85, 37.06)	37.14 (37.02, 37.26)	< 0.0001
Initial lactate level (mmol/L)	3.40 (3.18, 3.63)	3.02 (2.55, 3.49)	3.41 (2.92, 3.90)	3.14 (2.74, 3.54)	3.96 (3.53, 4.39)	0.02
Initial arterial PH	7.31 (7.30, 7.32)	7.32 (7.31, 7.34)	7.32 (7.30, 7.34)	7.29 (7.28, 7.31)	7.29 (7.27, 7.31)	< 0.01
Initial base excess	− 4.49 (− 4.94, − 4.03)	− 2.64 (− 3.46, − 1.83)	− 4.03 (− 5.03, − 3.03)	− 5.35 (− 6.23, − 4.47)	− 5.80 (− 6.67, − 4.92)	< 0.0001
ICU care unit, *n* (%)						< 0.0001
MICU/SICU	456 (36.0)	98 (7.7)	102 (8.1)	116 (9.2)	140 (11.1)	
MICU	388 (30.7)	106 (8.4)	106 (8.4)	94 (7.4)	82 (6.5)	
TSICU	157 (12.4)	28 (2.2)	40 (3.2)	40 (3.2)	49 (3.9)	
SICU	152 (12.0)	35 (2.8)	40 (3.2)	40 (3.2)	37 (2.9)	
CCU	61 (4.8)	22 (1.7)	16 (1.3)	16 (1.3)	7 (0.6)	
CVICU	36 (2.8)	17 (1.3)	10 (0.8)	7 (0.6)	2 (0.2)	
NSICU	14 (1.1)	8 (0.6)	3 (0.2)	2 (0.2)	1 (0.1)	
Neuro intermediate	2 (0.2)	2 (0.2)	0	0	0	
Net fluid balance						
At 24 h	3793.66 (3569.69, 4017.63)	2225.7 (1869.73, 2581.67)	3129.04 (2758.5, 3499.57)	3984.94 (3552.04, 4417.84)	5842.11 (5322.67, 6361.56)	< 0.0001
Norepinephrine max. dose (µg/kg/min)	4.82 (4.6, 5.05)	3.67 (3.26, 4.08)	4.46 (4.06, 4.87)	4.93 (4.50, 5.36)	6.22 (5.73, 6.72)	< 0.0001
Fluid input day 1	5319.1 (5116.46, 5521.74)	3931.86 (3598.99, 4264.74)	4725.87 (4405.14, 5046.60)	5517.15 (5126.63, 5907.68)	7092.8 (6614.96, 7570.65)	< 0.0001
Urine output	1569.67 (1494.21, 1645.14)	1721.16 (1565.28, 1877.04)	1647.15 (1502.88, 1791.43)	1550.68 (1413.86, 1687.51)	1358.96 (1194.26, 1523.66)	0.01
Time from VP treatment to ICU administration (hours)	14.33 (12.02, 16.65)	21.19 (14.60, 27.77)	18.92 (13.14, 24.70)	10.42 (8.10, 12.75)	6.82 (5.17, 8.47)	< 0.0001
Time from first fluid resuscitation load to ICU administration (hours)	3.37 (2.83, 3.91)	6.39 (4.07, 8.70)	3.50 (2.64, 4.36)	2.74 (2.07, 3.41)	1.88 (1.53, 2.22)	< 0.0001

*CCU* Coronary Care Unit, *CVICU* Cardiac Vascular Intensive Care Unit, *CI* confidence interval, *DSI* diastolic shock index, *DBP* diastolic blood pressure, *HR* heart rate, *ICU* Intensive Care Unit, *LOS* length of stay, *MSI* modified shock index, *LODS* logistic organ dysfunction system, *MAP* mean arterial pressure, *MICU* Medical Intensive Care Unit, *NSICU* Neuro Surgical Intensive Care Unit, *OASIS* Oxford Acute Severity of Illness Score, *Q* quartile, *RRT* renal replacement therapy, *SI* shock index, *sbp* systolic blood pressure, *SOFA* Sequential Organ Failure Assessment, *SICU* Surgical Intensive Care Unit, *TSICU* Trauma Surgical Intensive Care Unit, *VPs* start of vasopressors

^a^ Analysis of covariance or logistic regression analysis

^b^ Continuous variables were presented as mean (95% confidence interval) (all such values)

### Associations between SI, MSI, and DSI measurement and mortality

The multivariable logistic regression analysis showed a significant association between pre-VP SI and 3-day mortality in patients with septic shock requiring vasopressors before the start of VP treatment in fully adjusted models (*P* for trend < 0.01) (Table 2). Compared with the patients in the lowest quartile, the ORs (95% CIs) across increasing pre-VP SI were 4.67 (1.64 to 16.83), 3.02 (1.01 to 11.16), and 8.00 (2.92 to 28.31), respectively. Similar positive associations were also observed between pre-VP DSI/MSI and 3-day mortality (all *P* for trend < 0.01) (Tables 3 and 4). The ORs (95% CIs) across increasing pre-VP DSI were 1 (reference), 3.22 (1.16 to 10.43), 4.46 (1.69 to 14.07), and 4.29 (1.64 to 13.48), respectively. The ORs (95% CIs) across increasing pre-VP MSI were 1 (reference), 4.74 (1.64 to 17.21), 5.42 (1.94 to 19.32), and 5.22 (1.88 to 18.58), respectively. However, no significant associations were observed for pre-VP SI, MSI, and DSI with in-hospital mortality after adjusting for confounding factors (all *P* for trend > 0.01), although there appeared to be an increase in the risk in the upper quartile (Tables 2, 3 and 4). The ORs (95% CIs) across increasing pre-VP SI were 1 (reference), 1.15 (0.75–1.76), 1.21 (0.79–1.86), and 1.39 (0.90–2.15), respectively.

**Table 2:** Associations between shock index (SI) and mortality among patients with septic shock before the start of vasopressor therapy

	Quartiles of pre-VPs SI				*P* for trend ^a^
	Q1	Q2	Q3	Q4	
*n*	316	317	315	318	
Range	0.33, 0.77	0.77, 0.93	0.93, 1.13	1.13, 2.82	
3-day mortality					
Crude model	Ref.	3.46 (1.67, 7.87)	2.55 (1.19, 5.93)	6.43 (3.26, 14.22)	< 0.0001
Adjusted model 1^†^	Ref.	3.83 (1.83, 8.77)	3.14 (1.45, 7.38)	8.39 (4.16, 18.87)	< 0.0001
Adjusted model 2^‡^	Ref.	4.67 (1.64, 16.83)	3.02 (1.01, 11.16)	8.00 (2.92, 28.31)	< 0.001
In-hospital mortality					
Crude model	Ref.	1.29 (0.88, 1.89)	1.28 (0.87, 1.89)	1.73 (1.20, 2.52)	< 0.01
Adjusted model 1^†^	Ref.	1.36 (0.92, 2.01)	1.48 (1.00, 2.21)	2.04 (1.39, 3.02)	< 0.001
Adjusted model 2^‡^	Ref.	1.15 (0.75, 1.76)	1.21 (0.79, 1.86)	1.39 (0.90, 2.15)	0.13

SI, shock index; Q, quartile; VPs, start of vasopressors

^a^ Multiple logistic regression analysis

^b^ Odds ratios (95% confidence interval) (all such values)

^†^ Adjusted for age, gender, race, and ICU care unit

^‡^ Adjusted for variables in model 1 and SOFA score day-1

**Table 3:** Associations between modified shock index (MSI) and mortality among patients with septic shock before the start of vasopressor therapy

	Quartiles of pre-VPs MSI				*P* for trend ^a^
	Q1	Q2	Q3	Q4	
*n*	316	317	316	317	
Range	0.44, 1.12	1.12, 1.37	1.37, 1.64	1.64, 5.78	
3-day mortality					
Crude model	Ref.	1.66 (0.83, 3.47)	2.63 (1.38, 5.28)	3.78 (2.05, 7.45)	< 0.0001
Adjusted model 1^†^	Ref.	1.72 (0.85, 3.61)	3.00 (1.56, 6.08)	4.75 (2.53, 9.50)	< 0.0001
Adjusted model 2^‡^	Ref.	3.22 (1.16, 10.43)	4.46 (1.69, 14.07)	4.29 (1.64, 13.48)	0.01
In-hospital mortality					
Crude model	Ref.	1.09 (0.74, 1.61)	1.61 (1.11, 2.35)	1.5 (1.03, 2.18)	0.01
Adjusted model 1^†^	Ref.	1.12 (0.75, 1.66)	1.76 (1.21, 2.58)	1.75 (1.19, 2.58)	< 0.01
Adjusted model 2^‡^	Ref.	1.15 (0.75, 1.78)	1.63 (1.07, 2.48)	1.16 (0.74, 1.81)	0.36

*MSI* modified shock index, *Q* quartile, *VPs* start of vasopressors

^a^ Multiple logistic regression analysis

^b^ Odds ratios (95% confidence interval) (all such values)

^†^ Adjusted for age, gender, race, and ICU care unit

^‡^ Adjusted for variables in model 1 and SOFA score day-1

**Table 4:** Associations between diastolic shock index (DSI) and mortality among patients with septic shock before the start of vasopressor therapy

	Quartiles of pre-VPs DSI				*P* for trend ^a^
	Q1	Q2	Q3	Q4	
*n*	316	317	316	317	
Range	0.46, 1.35	1.35, 1.65	1.65, 1.96	1.96, 11.9	
3-day mortality					
Crude model	Ref.	1.85 (0.93, 3.82)	2.27 (1.18, 4.62)	4 (2.18, 7.86)	< 0.0001
Adjusted model 1^†^	Ref.	1.89 (0.94, 3.93)	2.49 (1.28, 5.11)	4.98 (2.67, 9.91)	< 0.0001
Adjusted model 2^‡^	Ref.	4.74 (1.64, 17.21)	5.42 (1.94, 19.32)	5.22 (1.88, 18.58)	< 0.01
In-hospital mortality					
Crude model	Ref.	1.56 (1.06, 2.32)	1.72 (1.17, 2.54)	1.93 (1.32, 2.85)	< 0.01
Adjusted model 1^†^	Ref.	1.59 (1.07, 2.38)	1.83 (1.24, 2.73)	2.21 (1.49, 3.29)	< 0.001
Adjusted model 2^‡^	Ref.	1.86 (1.20, 2.91)	1.89 (1.22, 2.94)	1.55 (0.99, 2.46)	0.11

*DSI* diastolic shock index, *Q* quartile, *VPs* start of vasopressors

^a^ Multiple logistic regression analysis

^b^ Odds ratios (95% confidence interval) (all such values

^†^ Adjusted for age, gender, race, and ICU care unit

^‡^ Adjusted for variables in model 1 and SOFA score day-1

**Fig. 2:**
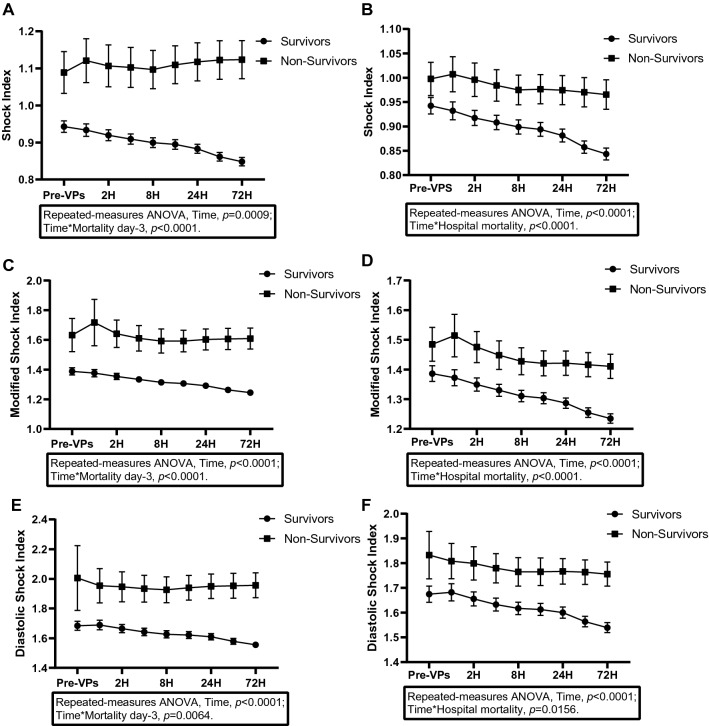
Time-course of shock index (SI), modified shock index (MSI), and diastolic shock index (DSI) for 3-day and in-hospital survivors and non-survivors. **A** Time-course of SI for 3-day survivors and non-survivors. **B** Time-course of SI for in-hospital survivors and non-survivors. **C** Time-course of MSI for 3-day survivors and non-survivors. **D** Time-course of MSI for in-hospital survivors and non-survivors. **E** Time-course of DSI for 3-day survivors and non-survivors. **F** Time-course of DSI for in-hospital survivors and non-survivors

There were significant differences in the time-course of SI, MSI, and DSI between survivors and non-survivors at 3-day mortality as well as in-hospital mortality among patients with septic shock requiring vasopressors (repeated-measures ANOVA, inter-subjects difference *P* < 0.001) (Fig. 2). The product of SI, MSI, and DSI remained significantly high in non-survivors.

The AUCs of pre-VPs SI, DSI, and MSI as well as SOFA score at day 1, Charlson comorbidity index, LODS OASIS, initial lactate level, and initial arterial PH were 0.746, 0.710, 0.732, 0.808, 0.739, 0.776, 0.793, 0.787, and 0.706 for 3-day mortality among patients with septic shock, respectively (Additional file 1: Figure S1). The AUCs of SI, DSI, and MSI at 1 h, 2 h, 4 h, 8 h, 12 h after VP treatment and SOFA score at day 1, Charlson comorbidity index, LODS OASIS, initial lactate level, and initial arterial PH are shown in Additional file 1: Figures S2–S6. In DeLong test, we found that the difference between the AUCs of pre-VPs SI/MSI and pre-VPs DSI was significant (*P* < 0.05). However, there were no difference between the AUCs of SI/MSI values and SOFA score at day 1 for 3-day mortality. Results of the ROC analysis indicated that the best cut-off values of SOFA score day-1 was 5.00 for 3-day mortality, resulting in 66.1% sensitivity and 85.5% specificity; the best cut-off value of pre-VPs SI was 0.97 for 3-day mortality, giving in 77.6% sensitivity and 77.1% specificity; the best cut-off value of pre-VPs MSI was 1.27 for 3-day mortality, giving in 68.4% sensitivity and 65.5% specificity (Additional file 1: Table S1). Cut-off values for SI, DSI, MSI after VP treatment for 3-day mortality and hospital mortality among patients with septic shock determined by ROC analysis and Youden’s index are shown in Additional file 1: Tables S2–S6.

## Discussion

In this study, we found that pre-VPs SI, MSI, and DSI values could identify patients with septic shock requiring vasopressors. There were significant differences in the time-course of SI, MSI, and DSI between survivors and non-survivors at 3-day/in-hospital mortality among patients with septic shock requiring vasopressors. The product of SI, MSI, and DSI remained significantly high in non-survivors.

In the United States, approximately 1.7 million cases of sepsis are registered each year, a trend that has been increasing annually. Sepsis causes almost 250,000 deaths annually, and it is the leading cause of death in non-cardiac ICUs [16, 17]. Notably, septic shock is a subset of sepsis associated with significantly higher mortality, and is characterized by persistent hypotension and persistently elevated lactate levels. Although the change in SOFA score is a robust mortality stratification tool, it is cumbersome to calculate in the clinic, and requires laboratory values that are not readily available for the quick screening of patients with potential septic shock [18]. Therefore, developing updated parameters or tools that could readily predict the outcomes of patients with septic shock, thereby guiding decisions and clinical management in the initial hours after the development of septic shock, is of paramount importance for reducing the mortality of patients with septic shock.

SI was first described in 1967 and provided an approximation of hemodynamic status in addition to traditional vital signs [19]. It has extensively been studied in ED-related studies, and could have potential value in patients admitted to the ED [11]. However, little is known regarding the role of SI in sepsis patients, especially in those with septic shock. A retrospective cohort of 2,524 adult patients with suspicion of sepsis compared the ability of SI to predict serum lactate levels ≥ 4 mmol/L compared to the ≥ 2 SIRS criteria and the modified SIRS (SIRS excluding white blood count) [12]. This large-scale cohort study showed that the positive predictive value of SI, SIRS, and modified SIRS in predicting both hyperlactatemia and 28-day mortality was limited. Compared to these findings, our study observed SI values could identify patients with septic shock requiring vasopressors who are at increased risk of early death. These discrepancies may be explained by the fact that our study only included patients with septic shock requiring vasopressors, a severe subtype of sepsis characterized by persistent hypotension.

MSI has been developed to incorporate MAP rather than SBP alone. A subsequent prospective longitudinal study of 9,860 adult trauma patients compared the predictive value of SI and MSI for in-hospital mortality [20]. The study showed that MSI < 0.7 and > 1.3 had higher odds of mortality than HR, SBP, DBP, and SI [20]. Loss of vascular tone is a key pathophysiological feature of septic shock in adults, and the combination of gradual diastolic hypertension and tachycardia may reflect more serious vasodilatory conditions. Based on this hypothesis, a previous study performed by Ospina-Tascón et al. showed that DSI pre-VPs and at several points after VP treatment initiation may be a very early identifier of patients with septic shock at high risk of death [10]. However, we did not observe significant differences between the AUCs of SI, MSI, and DSI values at different time points during the early phase of septic shock, including pre-VP treatment, and at 1 h, 2 h, 4 h, 8 h, and 12 h after VP treatment. Further studies are needed to determine and validate the exact values of SI, MSI, and DSI in predicting the mortality of patients with septic shock.

Our study has several strengths. First, to the best of our knowledge, this is the first study focusing on identifying patients with septic shock requiring vasopressors who are at increased risk of early death using a simple index that could help guide clinicians in the early phase of the disease. Second, in our study, we included 1,266 patients with septic shock requiring vasopressors; this large sample size provided sufficient reliability and effectiveness to evaluate very early mortality for patients with septic shock. Third, we evaluated time-course differences in SI, MSI, and DSI at different time point between 3-day/in-hospital septic shock requiring vasopressors survivors and non-survivors and performed comparisons between AUC for SI, MSI, DSI, SOFA score, Charlson comorbidity index, LODS and OASIS using DeLong method.

Our study has several limitations as well. First, this study is lack on information on the pre-ICU status of all included patients. Interventions in the ED or other departments may have impacted the ICU status and outcomes, but we could not perform the related analyses before admission to the ICU of all patients. Moreover, the MIMIC-IV database lacks information on patients admitted to the pediatric ICU and therefore we could not evaluate the roles of SI and pediatric-adjusted SI in predicting the mortality of septic shock in children. What’s more, the MIMIC database is based on a single-center study, and the findings may not be generalizable. Further prospective, large-scale, multicenter cohort studies are needed to confirm our results.

## Conclusions

Our study found that pre-vasopressor SI, MSI, and DSI could identify patients with septic shock requiring vasopressors who are at increased risk of early death. Of these easy-to-acquire values, SI and MSI show a comparatively better performance to risk stratify patients with septic shock, which can be used to guide clinical practice. Further external prospective and multicenter cohort studies will provide further validation and insight into these findings and potential causal mechanisms.

## Supplementary Information

**Additional file 1: Figure S1.** Receiver operating characteristic (ROC) curves at pre-VPs to predict 3-day and in-hospital mortality. **Table S1.** Cut-off values for SI, DSI, MSI at pre-VPs, Charlson comorbidity index, LODS score, OASIS score, and SOFA score, initial lactate level, and initial arterial PH as determined by ROC analysis and Youden’s index. **Figure S2.** Receiver operating characteristic (ROC) curves at 1 hour to predict 3-day and in-hospital mortality. **Table S2.** Cut-off values for SI, DSI, and MSI at 1 hour, Charlson comorbidity index, LODS score, OASIS score, and SOFA score, initial lactate level, and initial arterial PH as determined by ROC analysis and Youden’s index. **Figure S3**. Receiver operating characteristic (ROC) curves at 2 hour to predict 3-day and in-hospital mortality. **Table S3.** Cut-off values for SI, DSI, and MSI at 2 hour, Charlson comorbidity index, LODS score, OASIS score, and SOFA score, initial lactate level, and initial arterial PH as determined by ROC analysis and Youden’s index. **Table S4.** Cut-off values for SI, DSI, and MSI at 4 hour, Charlson comorbidity index, LODS score, OASIS score, and SOFA score, initial lactate level, and initial arterial PH as determined by ROC analysis and Youden’s index**. Figure S5.** Receiver operating characteristic (ROC) curves at 8 hour to predict 3-day and in-hospital mortality. **Table S5**. Cut-off values for SI, DSI, and MSI at 8 hour, Charlson comorbidity index, LODS score, OASIS score, and SOFA score, initial lactate level, and initial arterial PH as determined by ROC analysis and Youden’s index. **Figure S6.** Receiver operating characteristic (ROC) curves at 12 hour to predict 3-day and in-hospital mortality. **Table S6.** Cut-off values for SI, DSI, and MSI at 12 hour, Charlson comorbidity index, LODS score, OASIS score, and SOFA score, initial lactate level, and initial arterial PH as determined by ROC analysis and Youden’s index. **Table S7.** Characteristics of 1266 shock patients by quartiles of modified shock index before the start of vasopressor therapy. **Table S8.** Characteristics of 1266 shock patients by quartiles of diastolic shock index before the start of vasopressor therapy.

## Data Availability

The datasets used and/or analyzed during the current study are available from the corresponding author on reasonable request.

## Abbreviations

AUC: Area under the curve
CIs: Confidence intervals
DBP: Diastolic blood pressure
DSI: Diastolic shock index
ED: Emergency department
HR: Heart rate
ICU: Intensive care units
LODS: Logistic Organ Dysfunction Score
MAP: Mean arterial pressure
MIMIC-IV: Medical Information Mart for Intensive Care IV
MSI: Modified shock index
OASIS: Oxford Acute Severity of Illness Score
ORs: Odds ratios
Pre-VP: Pre-vasopressor
ROC: Receiver operating characteristic
RRT: Renal replacement therapy
SBP: Systolic blood pressure
SI: Shock index
SOFA: Sequential Organ Failure Assessment
STROBE: Strengthening the Reporting of Observational Studies in Epidemiology
