# Supplementary material for: Evaluation of SI, MSI and DSI for very early (3-day) mortality in patients with septic shock
Source: Eur J Med Res. 2022 Nov 3;27:227. doi: 10.1186/s40001-022-00857-y (PMC9632117; doi:10.1186/s40001-022-00857-y)
Supplement: Supplementary file 1 — Additional file 1: Figure S1. Receiver operating characteristic (ROC) curves at pre-VPs to predict 3-day and in-hospital mortality. Table S1. Cut-off values for SI, DSI, MSI at pre-VPs, Charlson comorbidity index, LODS score, OASIS score, and SOFA score, initial lactate level, and initial arterial PH as determined by ROC analysis and Youden’s index. Figure S2. Receiver operating characteristic (ROC) curves at 1 hour to predict 3-day and in-hospital mortality. Table S2. Cut-off values for SI, DSI, and MSI at 1 hour, Charlson comorbidity index, LODS score, OASIS score, and SOFA score, initial lactate level, and initial arterial PH as determined by ROC analysis and Youden’s index. Figure S3. Receiver operating characteristic (ROC) curves at 2 hour to predict 3-day and in-hospital mortality. Table S3. Cut-off values for SI, DSI, and MSI at 2 hour, Charlson comorbidity index, LODS score, OASIS score, and SOFA score, initial lactate level, and initial arterial PH as determined by ROC analysis and Youden’s index. Table S4. Cut-off values for SI, DSI, and MSI at 4 hour, Charlson comorbidity index, LODS score, OASIS score, and SOFA score, initial lactate level, and initial arterial PH as determined by ROC analysis and Youden’s index. Figure S5. Receiver operating characteristic (ROC) curves at 8 hour to predict 3-day and in-hospital mortality. Table S5. Cut-off values for SI, DSI, and MSI at 8 hour, Charlson comorbidity index, LODS score, OASIS score, and SOFA score, initial lactate level, and initial arterial PH as determined by ROC analysis and Youden’s index. Figure S6. Receiver operating characteristic (ROC) curves at 12 hour to predict 3-day and in-hospital mortality. Table S6. Cut-off values for SI, DSI, and MSI at 12 hour, Charlson comorbidity index, LODS score, OASIS score, and SOFA score, initial lactate level, and initial arterial PH as determined by ROC analysis and Youden’s index. Table S7. Characteristics of 1266 shock patients by quartiles of modified shock ind [file 40001_2022_857_MOESM1_ESM.pdf]

Supplemental Digital Content (SDC)

Effectiveness of SI, MSI and DSI at predicting very early (3-day) mortality in patients with septic shock

Tie-Ning Zhang[1#], Peng-Hui Hao[1#], Shan-Yan Gao[2, 3], Chun-Feng Liu[1], Ni Yang[1]

1. Department of Pediatrics, Shengjing Hospital of China Medical University, Shenyang, China. (T.N.Z., P.H.H., C.F.L, N.Y.)

2. Department of Clinical Epidemiology, Shengjing Hospital of China Medical University, Shenyang, China. (S.Y.G.)

3. Clinical Research Center, Shengjing Hospital of China Medical University, Shenyang, China. (S.Y.G.)

[#] These authors contributed equally to this work.

Correspondence to:

Ni Yang, M.D, Ph.D.

Department of Pediatrics, PICU

Shengjing Hospital of China Medical University

Address: No. 36, San Hao Street, Shenyang, Liaoning 110004, P. R. China

E-mail: yangni616@hotmail.com

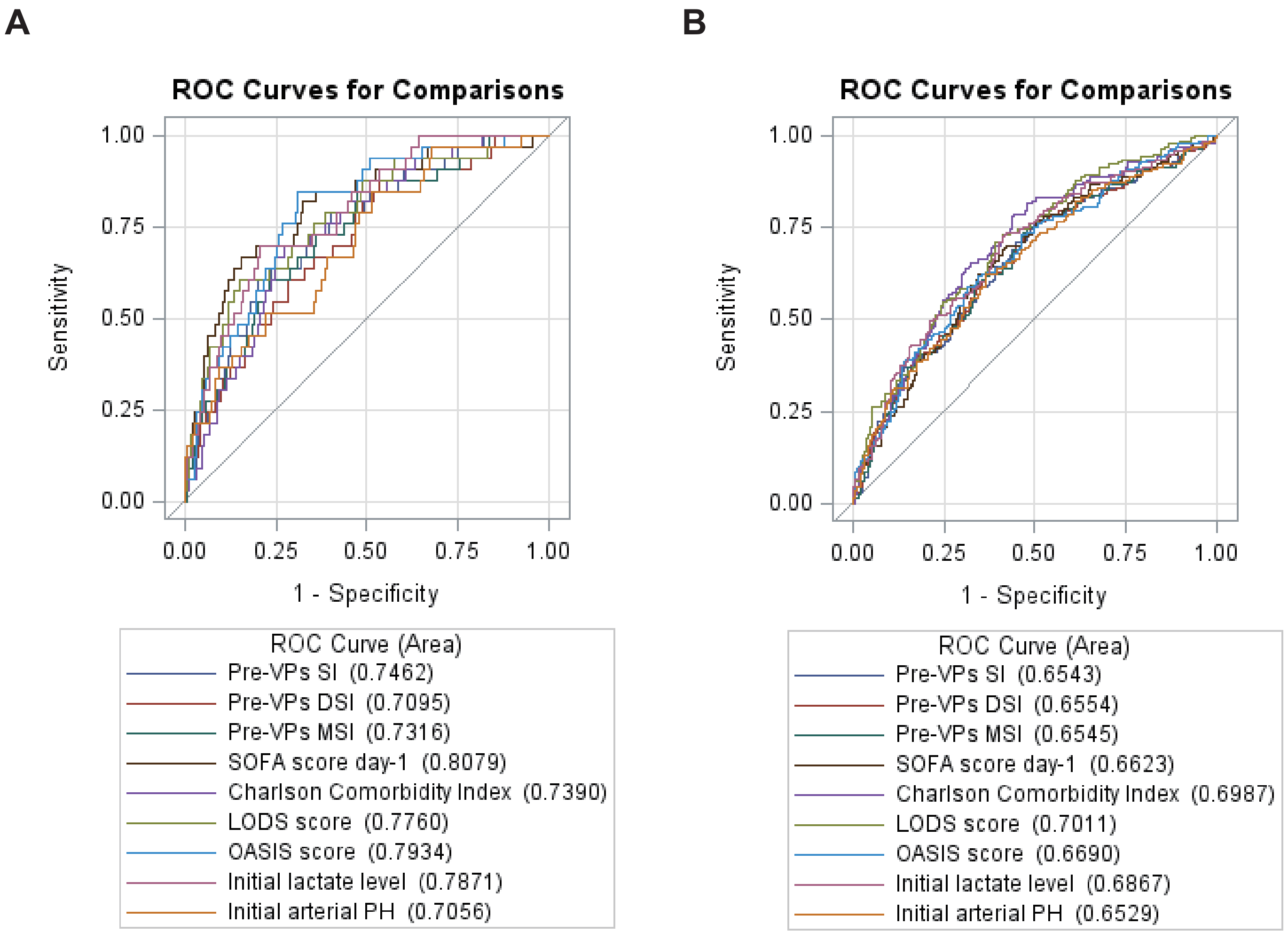

AUC – ROC (Pre-VPs): mortality day-3

Variables	AUC	Error tip.	p	95% CI	
				Lower Limit	Upper Limit
Pre-VPs SI	0.746	0.043	0.003	0.663	0.830
Pre-VPs DSI	0.710	0.045	0.100	0.621	0.798
Pre-VPs MSI	0.732	0.045	0.018	0.644	0.819
SOFA score day-1	0.808	0.042	<0.0001	0.727	0.889
Charlson Comorbidity Index	0.739	0.040	0.009	0.661	0.817
LODS score	0.776	0.043	<0.0001	0.691	0.861
OASIS score	0.793	0.037	<0.0001	0.721	0.866
Initial lactate level	0.787	0.038	<0.0001	0.714	0.861
Initial arterial PH	0.706	0.046	0.0126	0.616	0.795

AUC – ROC (Pre-VPs): hospital mortality

Variables	AUC	Error tip.	p	95% CI	
				Lower Limit	Upper Limit
Pre-VPs SI	0.654	0.027	0.496	0.601	0.707
Pre-VPs DSI	0.655	0.027	0.368	0.602	0.708
Pre-VPs MSI	0.655	0.027	0.461	0.602	0.707
SOFA score day-1	0.662	0.026	0.011	0.611	0.714
Charlson Comorbidity Index	0.699	0.025	<0.0001	0.649	0.748
LODS score	0.701	0.025	<0.0001	0.652	0.750
OASIS score	0.669	0.026	<0.001	0.617	0.721
Initial lactate level	0.687	0.026	<0.0001	0.636	0.738
Initial arterial PH	0.653	0.027	0.259	0.600	0.706

Delong test (Pre-VPs): mortality day-3

Variables	Estimate	Standard Error	95% CI		Chi-Square	p
			Lower Limit	Upper Limit		
**Pre-VPs SI - Pre-VPs DSI**	0.037	0.017	0.004	0.069	4.844	**0.028**
Pre-VPs SI - Pre-VPs MSI	0.015	0.012	-0.009	0.038	1.536	0.215
Pre-VPs SI - SOFA score day-1	-0.062	0.040	-0.140	0.017	2.351	0.125
Pre-VPs SI - Charlson Comorbidity Index	0.007	0.034	-0.058	0.073	0.047	0.829
Pre-VPs SI - LODS score	-0.030	0.034	-0.097	0.037	0.760	0.384
Pre-VPs SI - OASIS score	-0.047	0.033	-0.113	0.018	2.005	0.157
Pre-VPs SI - Initial lactate level	-0.041	0.042	-0.123	0.041	0.951	0.329
Pre-VPs SI - Initial arterial PH	0.041	0.042	-0.042	0.123	0.934	0.334
**Pre-VPs DSI - Pre-VPs MSI**	-0.022	0.009	-0.040	-0.004	5.550	**0.019**
**Pre-VPs DSI - SOFA score day-1**	-0.098	0.042	-0.180	-0.017	5.578	**0.018**
Pre-VPs DSI - Charlson Comorbidity Index	-0.030	0.028	-0.084	0.026	1.106	0.293
**Pre-VPs DSI - LODS score**	-0.066	0.033	-0.130	-0.003	4.173	**0.041**
**Pre-VPs DSI - OASIS score**	-0.084	0.034	-0.150	-0.018	6.210	**0.013**
**Pre-VPs DSI - Initial lactate level**	-0.078	0.039	-0.155	0.000	3.875	**0.049**
Pre-VPs DSI - Initial arterial PH	0.004	0.035	-0.064	0.072	0.013	0.910
Pre-VPs MSI - SOFA score day-1	-0.076	0.041	-0.156	0.004	3.477	0.062
Pre-VPs MSI - Charlson Comorbidity Index	-0.007	0.031	-0.068	0.053	0.057	0.811
Pre-VPs MSI - LODS score	-0.044	0.033	-0.109	0.021	1.791	0.181
Pre-VPs MSI - OASIS score	-0.062	0.034	-0.128	0.004	3.368	0.067
Pre-VPs MSI - Initial lactate level	-0.056	0.041	-0.135	0.024	1.862	0.172
Pre-VPs MSI - Initial arterial PH	0.026	0.039	-0.050	0.102	0.455	0.500
SOFA score day-1 - Charlson Comorbidity Index	0.069	0.047	-0.023	0.161	2.164	0.141
SOFA score day-1 - LODS score	0.032	0.031	-0.029	0.093	1.063	0.303
SOFA score day-1 - OASIS score	0.014	0.028	-0.040	0.069	0.274	0.601
SOFA score day-1 - Initial lactate level	0.021	0.047	-0.071	0.112	0.198	0.656
**SOFA score day-1 - Initial arterial PH**	0.102	0.044	0.017	0.188	5.519	**0.019**
Charlson Comorbidity Index - LODS score	-0.037	0.038	-0.112	0.038	0.941	0.332
Charlson Comorbidity Index - OASIS score	-0.054	0.039	-0.132	0.023	1.917	0.166
Charlson Comorbidity Index - Initial lactate level	-0.048	0.039	-0.125	0.029	1.506	0.220
Charlson Comorbidity Index - Initial arterial PH	0.033	0.042	-0.049	0.116	0.632	0.427
LODS score - OASIS score	-0.018	0.020	-0.056	0.021	0.777	0.378
LODS score - Initial lactate level	-0.011	0.043	-0.095	0.073	0.067	0.796
LODS score - Initial arterial PH	0.070	0.037	-0.002	0.143	3.599	0.058
OASIS score - Initial lactate level	0.006	0.039	-0.069	0.082	0.027	0.870
**OASIS score - Initial arterial PH**	0.088	0.035	0.019	0.156	6.294	**0.012**
**Initial lactate level - Initial arterial PH**	0.082	0.033	0.017	0.146	6.117	**0.013**

Delong test (Pre-VPs): hospital mortality

Variables	Estimate	Standard Error	95% CI		Chi-Square	p
			Lower Limit	Upper Limit		
Pre-VPs SI - Pre-VPs DSI	-0.001	0.003	-0.007	0.005	0.139	0.710
Pre-VPs SI - Pre-VPs MSI	0.000	0.002	-0.005	0.004	0.011	0.916
Pre-VPs SI - SOFA score day-1	-0.008	0.013	-0.033	0.017	0.407	0.524
**Pre-VPs SI - Charlson Comorbidity Index**	-0.044	0.020	-0.083	-0.006	5.040	**0.025**
**Pre-VPs SI - LODS score**	-0.047	0.020	-0.087	-0.007	5.273	**0.022**
Pre-VPs SI - OASIS score	-0.015	0.016	-0.047	0.017	0.807	0.369
Pre-VPs SI - Initial lactate level	-0.033	0.017	-0.066	0.001	3.533	0.060
Pre-VPs SI - Initial arterial PH	0.001	0.007	-0.013	0.015	0.035	0.852
Pre-VPs DSI - Pre-VPs MSI	0.001	0.002	-0.004	0.005	0.147	0.701
Pre-VPs DSI - SOFA score day-1	-0.007	0.013	-0.032	0.018	0.292	0.589
**Pre-VPs DSI - Charlson Comorbidity Index**	-0.043	0.020	-0.083	-0.004	4.578	**0.032**
**Pre-VPs DSI - LODS score**	-0.046	0.020	-0.086	-0.006	5.027	**0.025**
Pre-VPs DSI - OASIS score	-0.014	0.017	-0.046	0.019	0.667	0.414
Pre-VPs DSI - Initial lactate level	-0.031	0.018	-0.066	0.003	3.188	0.074
Pre-VPs DSI - Initial arterial PH	0.002	0.008	-0.013	0.018	0.093	0.761
**Pre-VPs MSI - SOFA score day-1**	-0.008	0.013	-0.033	0.017	0.384	0.536
**Pre-VPs MSI - Charlson Comorbidity Index**	-0.044	0.020	-0.083	-0.005	4.901	**0.027**
Pre-VPs MSI - LODS score	-0.047	0.020	-0.087	-0.007	5.211	**0.022**
Pre-VPs MSI - OASIS score	-0.015	0.017	-0.047	0.018	0.767	0.381
Pre-VPs MSI - Initial lactate level	-0.032	0.017	-0.066	0.002	3.452	0.063
Pre-VPs MSI - Initial arterial PH	0.002	0.007	-0.013	0.016	0.045	0.832
SOFA score day-1 - Charlson Comorbidity Index	-0.036	0.022	-0.079	0.006	2.799	0.094
**SOFA score day-1 - LODS score**	-0.039	0.017	-0.073	-0.005	4.969	**0.026**
SOFA score day-1 - OASIS score	-0.007	0.015	-0.036	0.023	0.191	0.662
SOFA score day-1 - Initial lactate level	-0.024	0.017	-0.058	0.009	2.028	0.154
SOFA score day-1 - Initial arterial PH	0.009	0.012	-0.015	0.034	0.584	0.445
Charlson Comorbidity Index - LODS score	-0.002	0.026	-0.053	0.048	0.009	0.924
Charlson Comorbidity Index - OASIS score	0.030	0.024	-0.017	0.077	1.545	0.214
Charlson Comorbidity Index - Initial lactate level	0.012	0.023	-0.033	0.057	0.269	0.604
**Charlson Comorbidity Index - Initial arterial PH**	0.046	0.021	0.005	0.086	4.878	**0.027**
**LODS score - OASIS score**	0.032	0.015	0.004	0.061	4.893	**0.027**
LODS score - Initial lactate level	0.014	0.024	-0.032	0.061	0.363	0.547
**LODS score - Initial arterial PH**	0.048	0.021	0.008	0.089	5.422	**0.020**
OASIS score - Initial lactate level	-0.018	0.021	-0.059	0.023	0.721	0.396
OASIS score - Initial arterial PH	0.016	0.016	-0.015	0.047	1.003	0.317
**Initial lactate level - Initial arterial PH**	0.034	0.017	0.001	0.066	4.142	**0.042**

Supplementary Figure 1. Receiver operating characteristic (ROC) curves at Pre-VPs to predict 3-day and in-hospital mortality.

Supplementary Table 1. Cut-off values for SI, DSI, MSI at pre-VPs, Charlson Comorbidity Index, LODS score, OASIS score, and SOFA score, initial lactate level, and initial arterial PH as determined by ROC analysis and Youden's index

	SI	DSI	MSI	Charlson Comorbidity Index	LODS	OASIS	SOFA	Initial lactate level	Initial arterial PH
**3-day mortality**									
**Cut-off**	**0.97**	**2.00**	**1.27**	**7.00**	**9.00**	**46.00**	**5.00**	**7.34**	**1.40**
Spec.	60.9%	62.7%	65.5%	76.4%	74.5%	71.8%	85.5%	62.5%	87.9%
Sens.	76.6%	67.4%	68.4%	52.7%	70.1%	73.1%	66.1%	75.4%	53.8%
PPV	95.4%	95.0%	95.4%	95.9%	96.7%	96.5%	98.8%	95.0%	97.7%
NPV	19.8%	15.5%	16.5%	13.3%	19.2%	20.3%	12.6%	21.2%	16.5%
**In-hospital mortality**									
**Cut-off**	**0.69**	**1.67**	**1.76**	**5.00**	**6.00**	**33.00**	**8.00**	**7.35**	**1.80**
Spec.	77.1%	77.8%	75.4%	60.3%	76.8%	78.8%	69.6%	60.9%	76.4%
Sens.	44.8%	43.9%	45.5%	64.4%	58.2%	53.9%	57.8%	64.2%	54.2%
PPV	86.5%	86.6%	85.8%	84.1%	89.1%	89.2%	87.4%	81.7%	86.3%
NPV	30.0%	29.8%	29.8%	34.2%	36.0%	34.4%	31.2%	38.6%	37.7%

Adjusted for age, gender, race, and ICU care unit.

DSI, Diastolic Shock Index; MSI, Modified Shock Index; NPV, negative predictive value; LODS, Logistic Organ Dysfunction System; OASIS, Oxford Acute Severity of Illness Score; PPV, positive predictive value; ROC, receiver operating characteristic; SI, Shock Index; SOFA, Sequential Organ Failure Assessment; Sens., sensitivity; Spec., specificity; VPs, Start of Vasopressors.

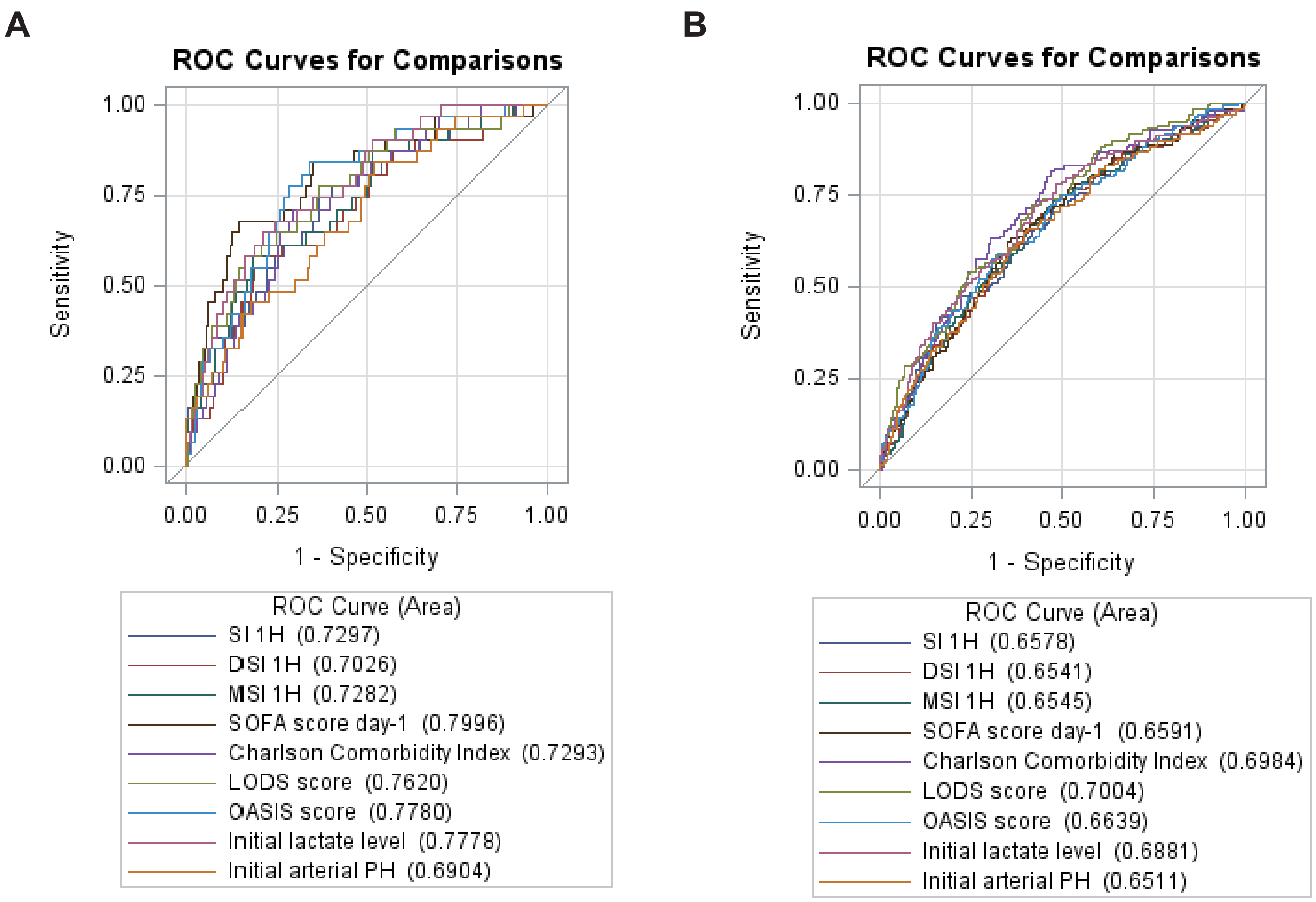

AUC – ROC (1 hour): mortality day-3

Variables	AUC	Error tip.	p	95% CI	
				Lower Limit	Upper Limit
SI 1H	0.730	0.047	0.002	0.637	0.822
DSI 1H	0.703	0.049	0.048	0.606	0.799
MSI 1H	0.728	0.048	0.002	0.635	0.822
SOFA score day-1	0.800	0.045	<0.0001	0.712	0.887
Charlson Comorbidity Index	0.729	0.042	0.005	0.646	0.812
LODS score	0.762	0.046	<0.001	0.673	0.851
OASIS score	0.778	0.039	<0.001	0.701	0.855
Initial lactate level	0.778	0.040	<0.0001	0.699	0.857
Initial arterial PH	0.690	0.048	0.028	0.597	0.784

AUC – ROC (1 hour): hospital mortality

Variables	AUC	Error tip.	p	95% CI	
				Lower Limit	Upper Limit
SI 1H	0.658	0.027	0.032	0.605	0.711
DSI 1H	0.654	0.027	0.100	0.601	0.707
MSI 1H	0.655	0.027	0.094	0.602	0.707
SOFA score day-1	0.659	0.027	0.009	0.607	0.711
Charlson Comorbidity Index	0.698	0.026	<0.0001	0.648	0.749
LODS score	0.700	0.025	<0.0001	0.651	0.750
OASIS score	0.664	0.027	<0.001	0.612	0.716
Initial lactate level	0.688	0.026	<0.0001	0.636	0.740
Initial arterial PH	0.651	0.027	0.364	0.597	0.705

Delong test (1 hour): mortality day-3

Variables	Estimate	Standard Error	95% CI		Chi-Square	p
			Lower Limit	Upper Limit		
SI 1H - DSI 1H	0.027	0.021	-0.015	0.069	1.600	0.206
SI 1H - MSI 1H	0.001	0.016	-0.030	0.033	0.009	0.927
SI 1H - SOFA score day-1	-0.070	0.044	-0.157	0.017	2.474	0.116
SI 1H - Charlson Comorbidity Index	0.000	0.044	-0.086	0.087	0.000	0.993
SI 1H - LODS score	-0.032	0.038	-0.107	0.043	0.714	0.398
SI 1H - OASIS score	-0.048	0.034	-0.115	0.018	2.020	0.155
SI 1H - Initial lactate level	-0.048	0.046	-0.138	0.042	1.106	0.293
SI 1H - Initial arterial PH	0.039	0.048	-0.055	0.134	0.664	0.415
**DSI 1H - MSI 1H**	-0.026	0.011	-0.048	-0.003	5.088	**0.024**
**DSI 1H - SOFA score day-1**	-0.097	0.044	-0.182	-0.012	4.979	**0.026**
DSI 1H - Charlson Comorbidity Index	-0.027	0.038	-0.101	0.047	0.499	0.480
DSI 1H - LODS score	-0.059	0.035	-0.128	0.009	2.906	0.088
**DSI 1H - OASIS score**	-0.075	0.033	-0.140	-0.011	5.250	**0.022**
DSI 1H - Initial lactate level	-0.075	0.041	-0.156	0.006	3.336	0.068
DSI 1H - Initial arterial PH	0.012	0.037	-0.061	0.085	0.109	0.742
MSI 1H - SOFA score day-1	-0.071	0.044	-0.157	0.014	2.696	0.101
MSI 1H - Charlson Comorbidity Index	-0.001	0.042	-0.082	0.080	0.001	0.979
MSI 1H - LODS score	-0.034	0.036	-0.105	0.037	0.868	0.352
MSI 1H - OASIS score	-0.050	0.033	-0.115	0.015	2.280	0.131
MSI 1H - Initial lactate level	-0.050	0.044	-0.135	0.036	1.297	0.255
MSI 1H - Initial arterial PH	0.038	0.043	-0.046	0.122	0.779	0.377
SOFA score day-1 - Charlson Comorbidity Index	0.070	0.051	-0.030	0.171	1.883	0.170
SOFA score day-1 - LODS score	0.038	0.033	-0.027	0.103	1.290	0.256
SOFA score day-1 - OASIS score	0.022	0.030	-0.036	0.080	0.532	0.466
SOFA score day-1 - Initial lactate level	0.022	0.050	-0.077	0.120	0.189	0.664
**SOFA score day-1 - Initial arterial PH**	0.109	0.046	0.020	0.198	5.748	**0.017**
Charlson Comorbidity Index - LODS score	-0.033	0.041	-0.113	0.048	0.638	0.424
Charlson Comorbidity Index - OASIS score	-0.049	0.042	-0.131	0.034	1.334	0.248
Charlson Comorbidity Index - Initial lactate level	-0.049	0.042	-0.131	0.034	1.323	0.250
Charlson Comorbidity Index - Initial arterial PH	0.039	0.046	-0.051	0.129	0.717	0.397
LODS score - OASIS score	-0.016	0.020	-0.056	0.024	0.615	0.433
LODS score - Initial lactate level	-0.016	0.045	-0.104	0.073	0.123	0.726
LODS score - Initial arterial PH	0.072	0.039	-0.005	0.148	3.394	0.065
OASIS score - Initial lactate level	0.000	0.040	-0.079	0.079	0.000	0.996
**OASIS score - Initial arterial PH**	0.088	0.036	0.017	0.158	5.933	**0.015**
**Initial lactate level - Initial arterial PH**	0.087	0.035	0.019	0.155	6.355	**0.012**

Delong test (1 hour): hospital mortality

Variables	Estimate	Standard Error	95% CI		Chi-Square	p
			Lower Limit	Upper Limit		
SI 1H - DSI 1H	0.004	0.008	-0.012	0.019	0.211	0.646
SI 1H - MSI 1H	0.003	0.006	-0.008	0.015	0.320	0.572
SI 1H - SOFA score day-1	-0.001	0.017	-0.034	0.031	0.006	0.938
SI 1H - Charlson Comorbidity Index	-0.041	0.023	-0.086	0.005	3.076	0.080
SI 1H - LODS score	-0.043	0.022	-0.086	0.001	3.631	0.057
SI 1H - OASIS score	-0.006	0.018	-0.042	0.030	0.114	0.736
SI 1H - Initial lactate level	-0.030	0.020	-0.069	0.008	2.387	0.122
SI 1H - Initial arterial PH	0.007	0.013	-0.019	0.032	0.257	0.612
DSI 1H - MSI 1H	0.000	0.005	-0.009	0.008	0.007	0.934
DSI 1H - SOFA score day-1	-0.005	0.016	-0.036	0.026	0.098	0.754
**DSI 1H - Charlson Comorbidity Index**	-0.044	0.022	-0.088	-0.001	3.929	**0.048**
**DSI 1H - LODS score**	-0.046	0.022	-0.088	-0.004	4.615	**0.032**
DSI 1H - OASIS score	-0.010	0.018	-0.045	0.026	0.297	0.586
DSI 1H - Initial lactate level	-0.034	0.020	-0.072	0.004	3.048	0.081
DSI 1H - Initial arterial PH	0.003	0.012	-0.019	0.026	0.069	0.793
MSI 1H - SOFA score day-1	-0.005	0.015	-0.034	0.025	0.090	0.764
**MSI 1H - Charlson Comorbidity Index**	-0.044	0.022	-0.087	-0.001	3.952	**0.047**
**MSI 1H - LODS score**	-0.046	0.021	-0.088	-0.004	4.600	**0.032**
MSI 1H - OASIS score	-0.009	0.018	-0.044	0.025	0.289	0.591
MSI 1H - Initial lactate level	-0.034	0.019	-0.071	0.003	3.194	0.074
MSI 1H - Initial arterial PH	0.003	0.011	-0.019	0.025	0.092	0.762
SOFA score day-1 - Charlson Comorbidity Index	-0.039	0.024	-0.085	0.007	2.796	0.095
**SOFA score day-1 - LODS score**	-0.041	0.018	-0.077	-0.006	5.134	**0.024**
SOFA score day-1 - OASIS score	-0.005	0.016	-0.036	0.026	0.095	0.758
SOFA score day-1 - Initial lactate level	-0.029	0.018	-0.064	0.006	2.626	0.105
SOFA score day-1 - Initial arterial PH	0.008	0.013	-0.018	0.034	0.364	0.546
Charlson Comorbidity Index - LODS score	-0.002	0.027	-0.055	0.052	0.005	0.942
Charlson Comorbidity Index - OASIS score	0.034	0.025	-0.015	0.084	1.860	0.173
Charlson Comorbidity Index - Initial lactate level	0.010	0.025	-0.038	0.058	0.177	0.674
**Charlson Comorbidity Index - Initial arterial PH**	0.047	0.022	0.004	0.090	4.655	**0.031**
**LODS score - OASIS score**	0.036	0.015	0.007	0.066	5.692	**0.017**
LODS score - Initial lactate level	0.012	0.025	-0.037	0.061	0.240	0.624
**LODS score - Initial arterial PH**	0.049	0.022	0.007	0.092	5.111	**0.024**
OASIS score - Initial lactate level	-0.024	0.022	-0.067	0.019	1.229	0.268
OASIS score - Initial arterial PH	0.013	0.017	-0.020	0.046	0.595	0.441
**Initial lactate level - Initial arterial PH**	0.037	0.018	0.003	0.071	4.446	**0.035**

Supplementary Figure 2. Receiver operating characteristic (ROC) curves at 1 hour to predict 3-day and in-hospital mortality.

Supplementary Table 2. Cut-off values for SI, DSI, and MSI at 1 hour, Charlson Comorbidity Index, LODS score, OASIS score, and SOFA score, initial lactate level, and initial arterial PH as determined by ROC analysis and Youden's index

	SI	DSI	MSI	Charlson Comorbidity Index	LODS	OASIS	SOFA	Initial lactate level	Initial arterial PH
**3-day mortality**									
**Cut-off**	**0.86**	**1.01**	**1.46**	**7.00**	**9.00**	**46.00**	**5.00**	**7.34**	**1.40**
Spec.	84.1%	66.4%	67.6%	76.4%	74.5%	71.8%	85.5%	62.5%	87.9%
Sens.	54.4%	63.5%	70.5%	52.7%	70.1%	73.1%	66.1%	75.4%	53.8%
PPV	97.3%	95.2%	95.8%	95.9%	96.7%	96.5%	98.8%	95.0%	97.7%
NPV	15.0%	14.8%	17.9%	13.3%	19.2%	20.3%	12.6%	21.2%	16.5%
**In-hospital mortality**									
**Cut-off**	**1.19**	**1.98**	**1.25**	**5.00**	**6.00**	**33.00**	**8.00**	**7.35**	**1.80**
Spec.	51.9%	56.7%	45.2%	60.3%	76.8%	78.8%	69.6%	60.9%	76.4%
Sens.	72.7%	65.2%	78.8%	64.4%	58.2%	53.9%	57.8%	64.2%	54.2%
PPV	83.0%	83.0%	82.3%	84.1%	89.1%	89.2%	87.4%	81.7%	86.3%
NPV	37.0%	33.5%	39.6%	34.2%	36.0%	34.4%	31.2%	38.6%	37.7%

Adjusted for age, gender, race, and ICU care unit.

DSI, Diastolic Shock Index; MSI, Modified Shock Index; NPV, negative predictive value; LODS, Logistic Organ Dysfunction System; OASIS, Oxford Acute Severity of Illness Score; PPV, positive predictive value; ROC, receiver operating characteristic; SI, Shock Index; SOFA, Sequential Organ Failure Assessment; Sens., sensitivity; Spec., specificity; VPs, Start of Vasopressors.

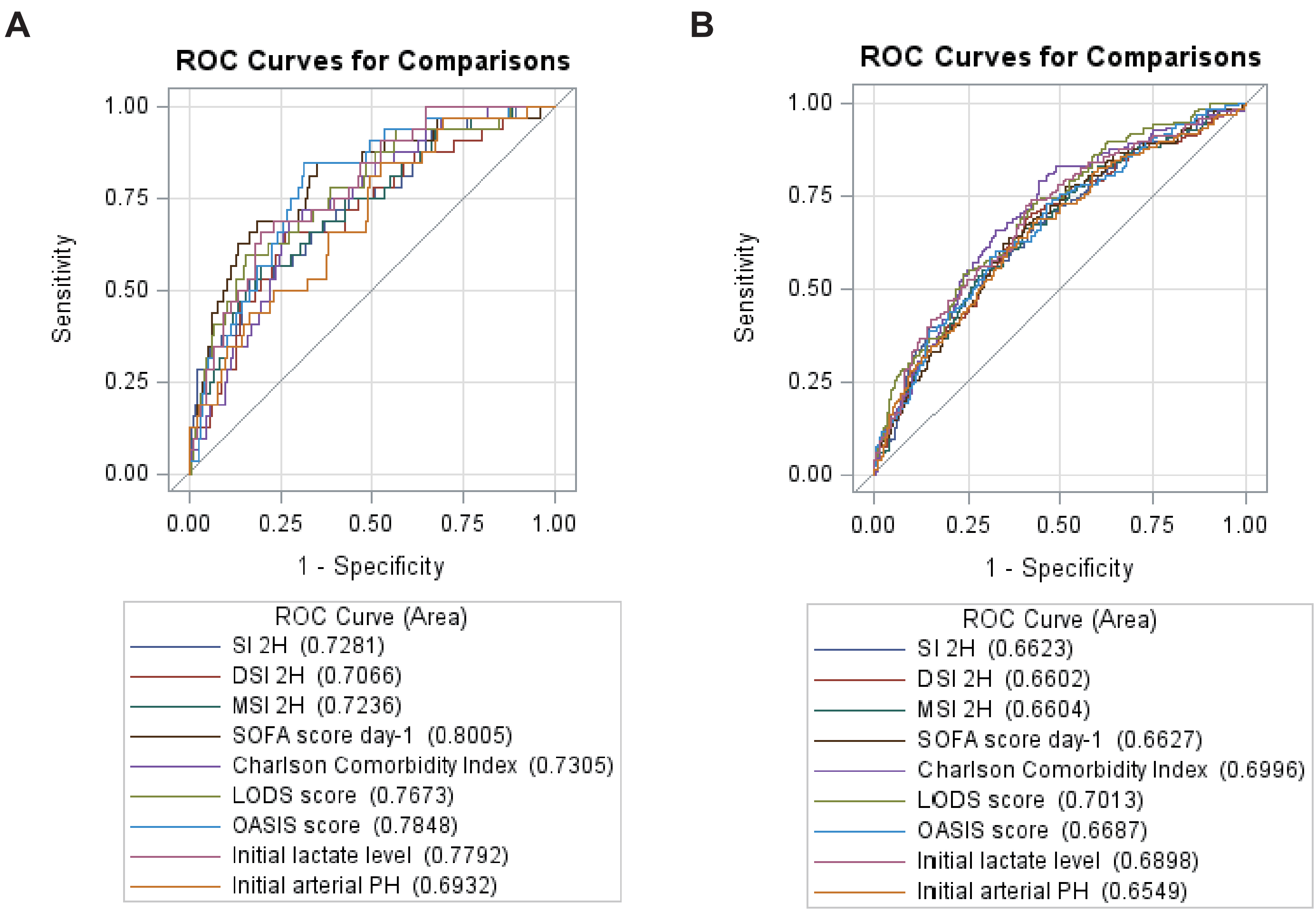

AUC – ROC (2 hour): mortality day-3

Variables	AUC	Error tip.	p	95% CI	
				Lower Limit	Upper Limit
SI 2H	0.728	0.048	0.001	0.634	0.822
DSI 2H	0.707	0.049	0.027	0.611	0.802
MSI 2H	0.724	0.047	0.002	0.631	0.816
SOFA score day-1	0.801	0.043	<0.0001	0.717	0.884
Charlson Comorbidity Index	0.731	0.041	0.008	0.651	0.810
LODS score	0.767	0.044	<0.001	0.681	0.854
OASIS score	0.785	0.038	<0.001	0.711	0.859
Initial lactate level	0.779	0.039	<0.0001	0.703	0.855
Initial arterial PH	0.693	0.046	0.028	0.603	0.784

AUC – ROC (2 hour): hospital mortality

Variables	AUC	Error tip.	p	95% CI	
				Lower Limit	Upper Limit
SI 2H	0.662	0.027	0.015	0.616	0.692
DSI 2H	0.660	0.027	0.021	0.608	0.684
MSI 2H	0.660	0.027	0.023	0.612	0.688
SOFA score day-1	0.663	0.026	0.010	0.644	0.718
Charlson Comorbidity Index	0.700	0.025	<0.0001	0.621	0.696
LODS score	0.701	0.025	<0.0001	0.694	0.763
OASIS score	0.669	0.027	<0.001	0.660	0.734
Initial lactate level	0.690	0.026	<0.0001	0.636	0.740
Initial arterial PH	0.655	0.027	0.299	0.597	0.705

Delong test (2 hour): mortality day-3

Variables	Estimate	Standard Error	95% CI		Chi-Square	p
			Lower Limit	Upper Limit		
SI 2H - DSI 2H	0.022	0.024	-0.025	0.068	0.840	0.359
SI 2H - MSI 2H	0.005	0.016	-0.027	0.036	0.079	0.778
SI 2H - SOFA score day-1	-0.072	0.049	-0.168	0.023	2.197	0.138
SI 2H - Charlson Comorbidity Index	-0.002	0.044	-0.089	0.084	0.003	0.957
SI 2H - LODS score	-0.039	0.043	-0.124	0.046	0.824	0.364
SI 2H - OASIS score	-0.057	0.039	-0.134	0.021	2.061	0.151
SI 2H - Initial lactate level	-0.051	0.046	-0.140	0.038	1.260	0.262
SI 2H - Initial arterial PH	0.035	0.049	-0.061	0.130	0.515	0.473
DSI 2H - MSI 2H	-0.017	0.013	-0.042	0.008	1.848	0.174
**DSI 2H - SOFA score day-1**	-0.094	0.044	-0.179	-0.008	4.639	**0.031**
DSI 2H - Charlson Comorbidity Index	-0.024	0.037	-0.096	0.048	0.430	0.512
DSI 2H - LODS score	-0.061	0.035	-0.130	0.008	2.997	0.083
**DSI 2H - OASIS score**	-0.078	0.034	-0.145	-0.012	5.283	**0.022**
DSI 2H - Initial lactate level	-0.073	0.040	-0.152	0.006	3.253	0.071
DSI 2H - Initial arterial PH	0.013	0.035	-0.056	0.083	0.142	0.706
MSI 2H - SOFA score day-1	-0.077	0.045	-0.164	0.011	2.969	0.085
MSI 2H - Charlson Comorbidity Index	-0.007	0.041	-0.087	0.074	0.028	0.867
MSI 2H - LODS score	-0.044	0.038	-0.119	0.032	1.296	0.255
MSI 2H - OASIS score	-0.061	0.035	-0.130	0.008	3.002	0.083
MSI 2H - Initial lactate level	-0.056	0.043	-0.140	0.028	1.684	0.194
MSI 2H - Initial arterial PH	0.030	0.043	-0.053	0.114	0.510	0.475
SOFA score day-1 - Charlson Comorbidity Index	0.070	0.049	-0.027	0.167	2.016	0.156
SOFA score day-1 - LODS score	0.033	0.032	-0.030	0.097	1.048	0.306
SOFA score day-1 - OASIS score	0.016	0.029	-0.040	0.072	0.303	0.582
SOFA score day-1 - Initial lactate level	0.021	0.049	-0.074	0.116	0.192	0.661
**SOFA score day-1 - Initial arterial PH**	0.107	0.045	0.020	0.195	5.758	**0.016**
Charlson Comorbidity Index - LODS score	-0.037	0.040	-0.116	0.042	0.839	0.360
Charlson Comorbidity Index - OASIS score	-0.054	0.041	-0.135	0.027	1.716	0.190
Charlson Comorbidity Index - Initial lactate level	-0.049	0.041	-0.130	0.032	1.395	0.238
Charlson Comorbidity Index - Initial arterial PH	0.037	0.044	-0.049	0.124	0.715	0.398
LODS score - OASIS score	-0.017	0.021	-0.058	0.023	0.703	0.402
LODS score - Initial lactate level	-0.012	0.045	-0.099	0.076	0.071	0.790
LODS score - Initial arterial PH	0.074	0.038	0.000	0.149	3.794	0.051
OASIS score - Initial lactate level	0.006	0.040	-0.073	0.084	0.019	0.890
**OASIS score - Initial arterial PH**	0.092	0.036	0.022	0.162	6.575	**0.010**
**Initial lactate level - Initial arterial PH**	0.086	0.034	0.019	0.153	6.284	**0.012**

Delong test (2 hour): hospital mortality

Variables	Estimate	Standard Error	95% CI		Chi-Square	p
			Lower Limit	Upper Limit		
SI 2H - DSI 2H	0.002	0.009	-0.015	0.019	0.060	0.807
SI 2H - MSI 2H	0.002	0.006	-0.009	0.013	0.124	0.725
SI 2H - SOFA score day-1	0.000	0.017	-0.034	0.033	0.001	0.980
SI 2H - Charlson Comorbidity Index	-0.037	0.023	-0.082	0.007	2.731	0.098
SI 2H - LODS score	-0.039	0.022	-0.082	0.004	3.187	0.074
SI 2H - OASIS score	-0.006	0.018	-0.042	0.029	0.122	0.727
SI 2H - Initial lactate level	-0.028	0.019	-0.065	0.010	2.076	0.150
SI 2H - Initial arterial PH	0.007	0.014	-0.020	0.035	0.289	0.591
DSI 2H - MSI 2H	0.000	0.005	-0.010	0.010	0.001	0.978
DSI 2H - SOFA score day-1	-0.003	0.017	-0.036	0.031	0.022	0.883
DSI 2H - Charlson Comorbidity Index	-0.039	0.023	-0.084	0.005	3.000	0.083
DSI 2H - LODS score	-0.041	0.021	-0.083	0.001	3.702	0.054
DSI 2H - OASIS score	-0.008	0.019	-0.046	0.029	0.196	0.658
DSI 2H - Initial lactate level	-0.030	0.020	-0.069	0.009	2.215	0.137
DSI 2H - Initial arterial PH	0.005	0.014	-0.023	0.034	0.138	0.710
MSI 2H - SOFA score day-1	-0.002	0.016	-0.034	0.030	0.021	0.885
MSI 2H - Charlson Comorbidity Index	-0.039	0.022	-0.083	0.004	3.122	0.077
MSI 2H - LODS score	-0.041	0.021	-0.082	0.000	3.765	0.052
MSI 2H - OASIS score	-0.008	0.018	-0.044	0.027	0.208	0.648
MSI 2H - Initial lactate level	-0.029	0.019	-0.067	0.008	2.420	0.120
MSI 2H - Initial arterial PH	0.005	0.014	-0.022	0.032	0.159	0.690
SOFA score day-1 - Charlson Comorbidity Index	-0.037	0.023	-0.081	0.007	2.685	0.101
**SOFA score day-1 - LODS score**	-0.039	0.018	-0.073	-0.004	4.738	**0.030**
SOFA score day-1 - OASIS score	-0.006	0.016	-0.036	0.025	0.146	0.702
SOFA score day-1 - Initial lactate level	-0.027	0.017	-0.061	0.007	2.411	0.121
SOFA score day-1 - Initial arterial PH	0.008	0.013	-0.017	0.033	0.386	0.534
Charlson Comorbidity Index - LODS score	-0.002	0.026	-0.053	0.050	0.004	0.951
Charlson Comorbidity Index - OASIS score	0.031	0.024	-0.017	0.079	1.607	0.205
Charlson Comorbidity Index - Initial lactate level	0.010	0.024	-0.036	0.056	0.174	0.677
**Charlson Comorbidity Index - Initial arterial PH**	0.045	0.021	0.003	0.086	4.457	**0.035**
**LODS score - OASIS score**	0.033	0.015	0.004	0.062	4.839	**0.028**
LODS score - Initial lactate level	0.012	0.024	-0.036	0.059	0.221	0.638
**LODS score - Initial arterial PH**	0.046	0.021	0.005	0.088	4.852	**0.028**
OASIS score - Initial lactate level	-0.021	0.021	-0.063	0.021	0.985	0.321
OASIS score - Initial arterial PH	0.014	0.016	-0.018	0.046	0.720	0.396
**Initial lactate level - Initial arterial PH**	0.035	0.017	0.002	0.068	4.274	**0.039**

Supplementary Figure 3. Receiver operating characteristic (ROC) curves at 2 hour to predict 3-day and in-hospital mortality.

Supplementary Table 3. Cut-off values for SI, DSI, and MSI at 2 hour, Charlson Comorbidity Index, LODS score, OASIS score, and SOFA score, initial lactate level, and initial arterial PH as determined by ROC analysis and Youden's index

	SI	DSI	MSI	Charlson Comorbidity Index	LODS	OASIS	SOFA	Initial lactate level	Initial arterial PH
**3-day mortality**									
**Cut-off**	**0.69**	**1.31**	**2.11**	**7.00**	**9.00**	**46.00**	**5.00**	**7.34**	**1.40**
Spec.	76.9%	67.6%	70.6%	76.4%	74.5%	71.8%	85.5%	62.5%	87.9%
Sens.	62.6%	65.1%	66.0%	52.7%	70.1%	73.1%	66.1%	75.4%	53.8%
PPV	96.6%	95.5%	96.0%	95.9%	96.7%	96.5%	98.8%	95.0%	97.7%
NPV	16.3%	15.5%	16.5%	13.3%	19.2%	20.3%	12.6%	21.2%	16.5%
**In-hospital mortality**									
**Cut-off**	**0.81**	**1.82**	**1.38**	**5.00**	**6.00**	**33.00**	**8.00**	**7.35**	**1.80**
Spec.	55.5%	63.0%	57.3%	76.4%	74.5%	71.8%	85.5%	62.5%	87.9%
Sens.	68.6%	60.4%	66.6%	60.3%	76.8%	78.8%	69.6%	60.9%	76.4%
PPV	83.4%	84.2%	83.6%	84.1%	89.1%	89.2%	87.4%	81.7%	86.3%
NPV	35.1%	32.8%	34.4%	34.2%	36.0%	34.4%	31.2%	38.6%	37.7%

Adjusted for age, gender, race, and ICU care unit.

DSI, Diastolic Shock Index; MSI, Modified Shock Index; NPV, negative predictive value; LODS, Logistic Organ Dysfunction System; OASIS, Oxford Acute Severity of Illness Score; PPV, positive predictive value; ROC, receiver operating characteristic; SI, Shock Index; SOFA, Sequential Organ Failure Assessment; Sens., sensitivity; Spec., specificity; VPs, Start of Vasopressors.

A

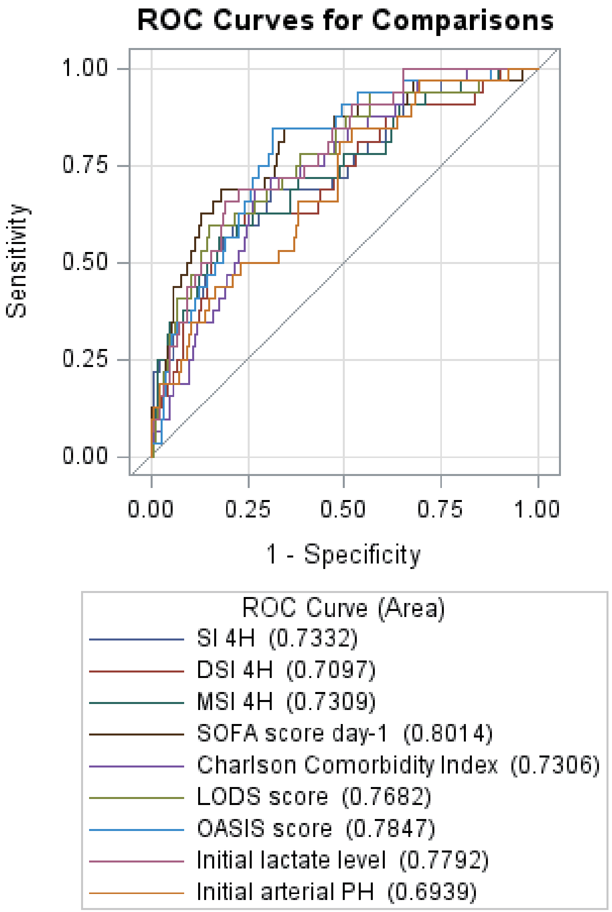

B

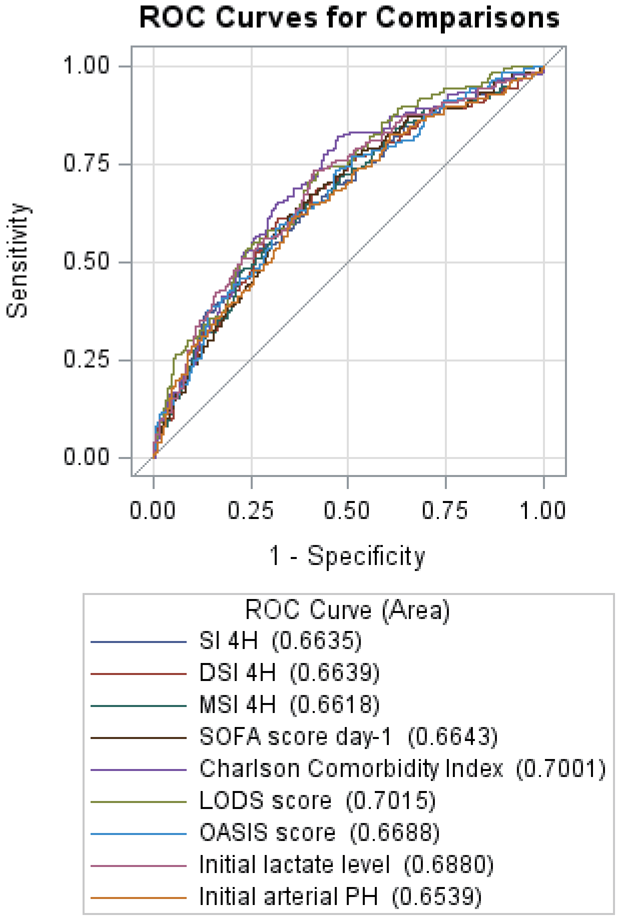

AUC – ROC (4 hour): mortality day-3

Variables	AUC	Error tip.	p	95% CI	
				Lower Limit	Upper Limit
SI 4H	0.733	0.049	<0.001	0.638	0.828
DSI 4H	0.710	0.050	0.008	0.611	0.808
MSI 4H	0.731	0.049	<0.001	0.634	0.828
SOFA score day-1	0.801	0.043	<0.0001	0.718	0.885
Charlson Comorbidity Index	0.731	0.041	0.008	0.651	0.810
LODS score	0.768	0.044	<0.001	0.681	0.855
OASIS score	0.785	0.038	<0.0001	0.711	0.859
Initial lactate level	0.779	0.039	<0.0001	0.703	0.855
Initial arterial PH	0.694	0.046	0.026	0.603	0.785

AUC – ROC (4 hour): hospital mortality

Variables	AUC	Error tip.	p	95% CI	
				Lower Limit	Upper Limit
SI 4H	0.664	0.027	0.015	0.611	0.716
DSI 4H	0.664	0.027	0.012	0.611	0.717
MSI 4H	0.662	0.027	0.023	0.609	0.714
SOFA score day-1	0.664	0.026	0.007	0.613	0.716
Charlson Comorbidity Index	0.700	0.025	<0.0001	0.650	0.750
LODS score	0.702	0.025	<0.0001	0.653	0.750
OASIS score	0.669	0.026	<0.001	0.617	0.720
Initial lactate level	0.688	0.026	<0.0001	0.637	0.739
Initial arterial PH	0.654	0.027	0.293	0.601	0.707

Delong test (4 hour): mortality day-3

Variables	Estimate	Standard Error	95% CI		Chi-Square	p
			Lower Limit	Upper Limit		
SI 4H - DSI 4H	0.024	0.019	-0.014	0.061	1.510	0.219
SI 4H - MSI 4H	0.002	0.015	-0.027	0.031	0.024	0.876
SI 4H - SOFA score day-1	-0.068	0.049	-0.164	0.027	1.968	0.161
SI 4H - Charlson Comorbidity Index	0.003	0.043	-0.082	0.088	0.004	0.952
SI 4H - LODS score	-0.035	0.042	-0.117	0.047	0.704	0.401
SI 4H - OASIS score	-0.052	0.039	-0.127	0.024	1.788	0.181
SI 4H - Initial lactate level	-0.046	0.045	-0.135	0.043	1.036	0.309
SI 4H - Initial arterial PH	0.039	0.048	-0.055	0.133	0.668	0.414
DSI 4H - MSI 4H	-0.021	0.011	-0.043	0.000	3.796	0.051
DSI 4H - SOFA score day-1	-0.092	0.047	-0.184	0.000	3.843	0.050
DSI 4H - Charlson Comorbidity Index	-0.021	0.042	-0.103	0.061	0.251	0.616
DSI 4H - LODS score	-0.059	0.038	-0.134	0.017	2.319	0.128
**DSI 4H - OASIS score**	-0.075	0.036	-0.146	-0.004	4.300	**0.038**
DSI 4H - Initial lactate level	-0.070	0.042	-0.152	0.013	2.737	0.098
DSI 4H - Initial arterial PH	0.016	0.039	-0.060	0.092	0.164	0.685
MSI 4H - SOFA score day-1	-0.071	0.048	-0.164	0.023	2.187	0.139
MSI 4H - Charlson Comorbidity Index	0.000	0.044	-0.086	0.087	0.000	0.995
MSI 4H - LODS score	-0.037	0.040	-0.116	0.042	0.856	0.355
MSI 4H - OASIS score	-0.054	0.038	-0.128	0.020	2.050	0.152
MSI 4H - Initial lactate level	-0.048	0.043	-0.133	0.036	1.253	0.263
MSI 4H - Initial arterial PH	0.037	0.045	-0.051	0.125	0.674	0.412
SOFA score day-1 - Charlson Comorbidity Index	0.071	0.049	-0.026	0.167	2.067	0.151
SOFA score day-1 - LODS score	0.033	0.032	-0.030	0.097	1.057	0.304
SOFA score day-1 - OASIS score	0.017	0.029	-0.039	0.073	0.343	0.558
SOFA score day-1 - Initial lactate level	0.022	0.048	-0.073	0.117	0.210	0.647
**SOFA score day-1 - Initial arterial PH**	0.108	0.045	0.020	0.195	5.765	**0.016**
Charlson Comorbidity Index - LODS score	-0.038	0.040	-0.116	0.041	0.872	0.350
Charlson Comorbidity Index - OASIS score	-0.054	0.041	-0.135	0.027	1.708	0.191
Charlson Comorbidity Index - Initial lactate level	-0.049	0.041	-0.129	0.032	1.401	0.237
Charlson Comorbidity Index - Initial arterial PH	0.037	0.044	-0.050	0.123	0.688	0.407
LODS score - OASIS score	-0.017	0.021	-0.057	0.024	0.644	0.422
LODS score - Initial lactate level	-0.011	0.044	-0.098	0.076	0.062	0.803
LODS score - Initial arterial PH	0.074	0.038	0.000	0.149	3.800	0.051
OASIS score - Initial lactate level	0.005	0.040	-0.073	0.084	0.019	0.891
**OASIS score - Initial arterial PH**	0.091	0.036	0.021	0.161	6.463	**0.011**
**Initial lactate level - Initial arterial PH**	0.085	0.034	0.019	0.152	6.280	**0.012**

Delong test (4 hour): hospital mortality

Variables	Estimate	Standard Error	95% CI		Chi-Square	p
			Lower Limit	Upper Limit		
SI 4H - DSI 4H	0.000	0.009	-0.019	0.018	0.002	0.968
SI 4H - MSI 4H	0.002	0.005	-0.009	0.012	0.101	0.751
SI 4H - SOFA score day-1	-0.001	0.017	-0.034	0.032	0.002	0.965
SI 4H - Charlson Comorbidity Index	-0.037	0.022	-0.080	0.007	2.742	0.098
SI 4H - LODS score	-0.038	0.022	-0.080	0.004	3.128	0.077
SI 4H - OASIS score	-0.005	0.018	-0.040	0.030	0.086	0.769
SI 4H - Initial lactate level	-0.025	0.019	-0.061	0.012	1.727	0.189
SI 4H - Initial arterial PH	0.010	0.014	-0.017	0.036	0.500	0.479
DSI 4H - MSI 4H	0.002	0.006	-0.009	0.013	0.138	0.710
DSI 4H - SOFA score day-1	0.000	0.018	-0.035	0.034	0.000	0.984
DSI 4H - Charlson Comorbidity Index	-0.036	0.023	-0.081	0.008	2.546	0.111
DSI 4H - LODS score	-0.038	0.021	-0.079	0.004	3.120	0.077
DSI 4H - OASIS score	-0.005	0.019	-0.043	0.033	0.064	0.800
DSI 4H - Initial lactate level	-0.024	0.020	-0.063	0.015	1.469	0.226
DSI 4H - Initial arterial PH	0.010	0.015	-0.020	0.040	0.427	0.513
MSI 4H - SOFA score day-1	-0.002	0.017	-0.035	0.030	0.022	0.882
MSI 4H - Charlson Comorbidity Index	-0.038	0.022	-0.081	0.005	3.069	0.080
MSI 4H - LODS score	-0.040	0.021	-0.081	0.001	3.584	0.058
MSI 4H - OASIS score	-0.007	0.018	-0.043	0.029	0.148	0.701
MSI 4H - Initial lactate level	-0.026	0.019	-0.063	0.010	2.006	0.157
MSI 4H - Initial arterial PH	0.008	0.014	-0.019	0.035	0.332	0.565
SOFA score day-1 - Charlson Comorbidity Index	-0.036	0.023	-0.080	0.008	2.527	0.112
**SOFA score day-1 - LODS score**	-0.037	0.018	-0.072	-0.003	4.485	**0.034**
SOFA score day-1 - OASIS score	-0.005	0.016	-0.035	0.026	0.084	0.771
SOFA score day-1 - Initial lactate level	-0.024	0.018	-0.058	0.011	1.846	0.174
SOFA score day-1 - Initial arterial PH	0.010	0.013	-0.015	0.036	0.628	0.428
Charlson Comorbidity Index - LODS score	-0.001	0.026	-0.053	0.050	0.003	0.956
Charlson Comorbidity Index - OASIS score	0.031	0.024	-0.016	0.079	1.654	0.199
Charlson Comorbidity Index - Initial lactate level	0.012	0.023	-0.034	0.058	0.265	0.607
**Charlson Comorbidity Index - Initial arterial PH**	0.046	0.021	0.005	0.088	4.767	**0.029**
**LODS score - OASIS score**	0.033	0.015	0.004	0.062	4.917	**0.027**
LODS score - Initial lactate level	0.014	0.024	-0.034	0.061	0.310	0.577
**LODS score - Initial arterial PH**	0.048	0.021	0.006	0.089	5.129	**0.024**
OASIS score - Initial lactate level	-0.019	0.021	-0.061	0.022	0.831	0.362
OASIS score - Initial arterial PH	0.015	0.016	-0.017	0.047	0.834	0.361
**Initial lactate level - Initial arterial PH**	0.034	0.017	0.002	0.067	4.208	**0.040**

Supplementary Figure 4. Receiver operating characteristic (ROC) curves at 4 hour to predict 3-day and in-hospital mortality.

Supplementary Table 4. Cut-off values for SI, DSI, and MSI at 4 hour, Charlson Comorbidity Index, LODS score, OASIS score, and SOFA score, initial lactate level, and initial arterial PH as determined by ROC analysis and Youden's index

	SI	DSI	MSI	Charlson Comorbidity Index	LODS	OASIS	SOFA	Initial lactate level	Initial arterial PH
**3-day mortality**									
**Cut-off**	**1.55**	**2.10**	**1.72**	**7.00**	**9.00**	**46.00**	**5.00**	**7.34**	**1.40**
Spec.	75.9%	71.3%	82.6%	76.4%	74.5%	71.8%	85.5%	62.5%	87.9%
Sens.	65.1%	64.9%	56.0%	52.7%	70.1%	73.1%	66.1%	75.4%	53.8%
PPV	96.6%	96.0%	97.1%	95.9%	96.7%	96.5%	98.8%	95.0%	97.7%
NPV	17.0%	16.1%	15.1%	13.3%	19.2%	20.3%	12.6%	21.2%	16.5%
**In-hospital mortality**									
**Cut-off**	**0.92**	**1.73**	**1.16**	**5.00**	**6.00**	**33.00**	**8.00**	**7.35**	**1.80**
Spec.	66.7%	67.3%	70.9%	60.3%	76.8%	78.8%	69.6%	60.9%	76.4%
Sens.	58.2%	56.3%	53.5%	64.4%	58.2%	53.9%	57.8%	64.2%	54.2%
PPV	85.0%	84.9%	85.8%	84.1%	89.1%	89.2%	87.4%	81.7%	86.3%
NPV	32.9%	32.1%	31.8%	34.2%	36.0%	34.4%	31.2%	38.6%	37.7%

Adjusted for age, gender, race, and ICU care unit.

DSI, Diastolic Shock Index; MSI, Modified Shock Index; NPV, negative predictive value; LODS, Logistic Organ Dysfunction System; OASIS, Oxford Acute Severity of Illness Score; PPV, positive predictive value; ROC, receiver operating characteristic; SI, Shock Index; SOFA, Sequential Organ Failure Assessment; Sens., sensitivity; Spec., specificity; VPs, Start of Vasopressors.

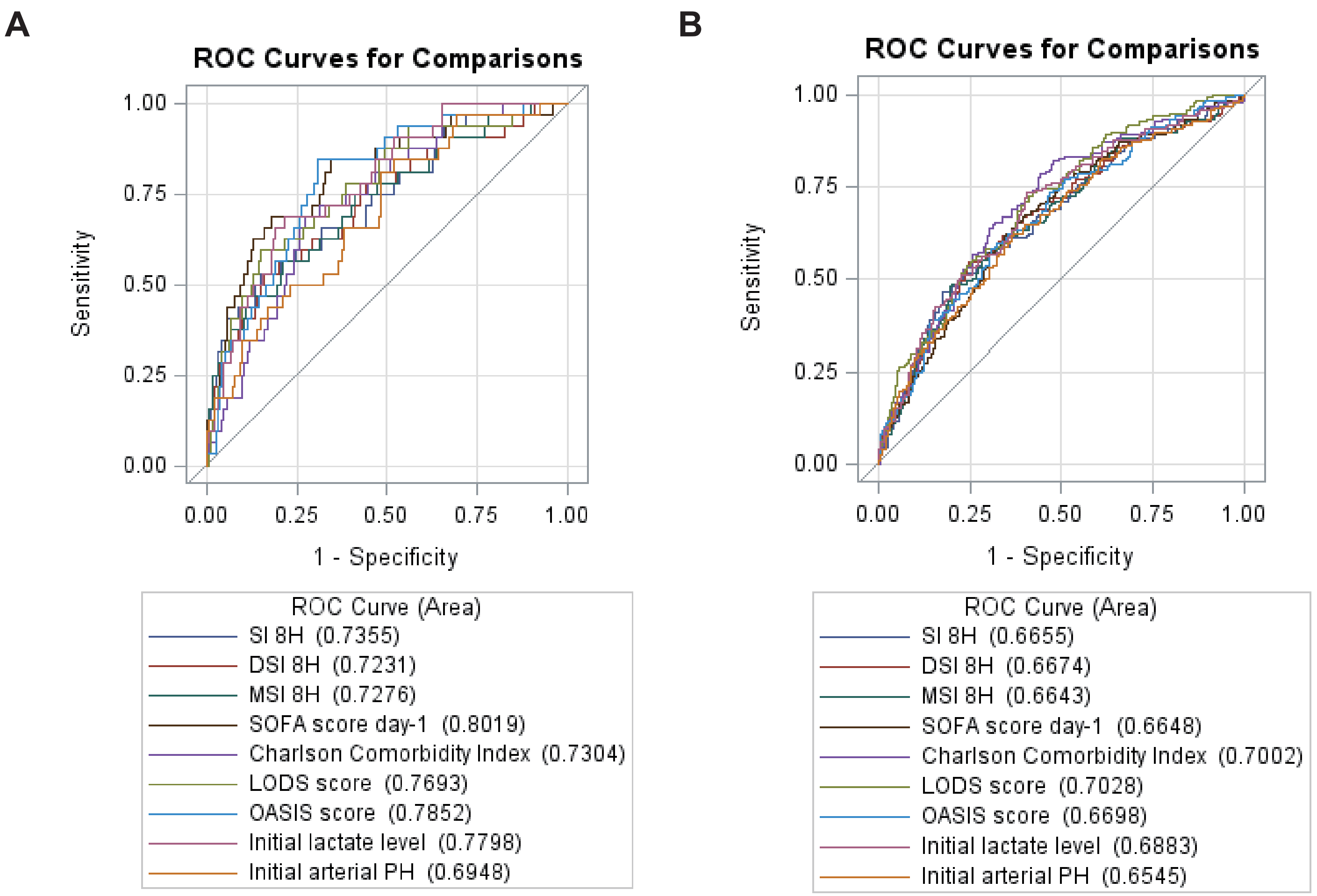

AUC – ROC (8 hour): mortality day-3

Variables	AUC	Error tip.	p	95% CI	
				Lower Limit	Upper Limit
SI 8H	0.736	0.049	<0.001	0.640	0.831
DSI 8H	0.723	0.050	0.003	0.625	0.821
MSI 8H	0.728	0.049	<0.001	0.631	0.824
SOFA score day-1	0.802	0.043	<0.0001	0.718	0.886
Charlson Comorbidity Index	0.730	0.041	0.008	0.651	0.810
LODS score	0.769	0.044	<0.001	0.683	0.856
OASIS score	0.785	0.038	<0.001	0.711	0.859
Initial lactate level	0.780	0.039	<0.0001	0.704	0.856
Initial arterial PH	0.695	0.046	0.025	0.604	0.785

AUC – ROC (8 hour): hospital mortality

Variables	AUC	Error tip.	p	95% CI	
				Lower Limit	Upper Limit
SI 8H	0.666	0.027	0.022	0.613	0.719
DSI 8H	0.667	0.027	0.005	0.614	0.721
MSI 8H	0.664	0.027	0.017	0.611	0.717
SOFA score day-1	0.665	0.026	0.006	0.613	0.716
Charlson Comorbidity Index	0.700	0.025	<0.0001	0.651	0.750
LODS score	0.703	0.025	<0.0001	0.654	0.751
OASIS score	0.670	0.026	<0.001	0.618	0.721
Initial lactate level	0.688	0.026	<0.0001	0.637	0.740
Initial arterial PH	0.655	0.027	0.280	0.601	0.708

Delong test (8 hour): mortality day-3

Variables	Estimate	Standard Error	95% CI		Chi-Square	p
			Lower Limit	Upper Limit		
SI 8H - DSI 8H	0.013	0.020	-0.027	0.052	0.387	0.534
SI 8H - MSI 8H	0.008	0.016	-0.023	0.039	0.253	0.615
SI 8H - SOFA score day-1	-0.066	0.048	-0.160	0.027	1.923	0.166
SI 8H - Charlson Comorbidity Index	0.005	0.043	-0.080	0.090	0.014	0.905
SI 8H - LODS score	-0.034	0.041	-0.114	0.046	0.680	0.410
SI 8H - OASIS score	-0.050	0.038	-0.124	0.024	1.741	0.187
SI 8H - Initial lactate level	-0.044	0.043	-0.128	0.039	1.079	0.299
SI 8H - Initial arterial PH	0.041	0.046	-0.050	0.132	0.769	0.381
DSI 8H - MSI 8H	-0.004	0.011	-0.027	0.018	0.155	0.693
DSI 8H - SOFA score day-1	-0.079	0.046	-0.169	0.011	2.932	0.087
DSI 8H - Charlson Comorbidity Index	-0.007	0.042	-0.089	0.075	0.031	0.861
DSI 8H - LODS score	-0.046	0.038	-0.120	0.028	1.492	0.222
DSI 8H - OASIS score	-0.062	0.036	-0.132	0.008	3.002	0.083
DSI 8H - Initial lactate level	-0.057	0.041	-0.138	0.024	1.893	0.169
DSI 8H - Initial arterial PH	0.028	0.037	-0.045	0.101	0.574	0.449
MSI 8H - SOFA score day-1	-0.074	0.047	-0.167	0.018	2.504	0.114
MSI 8H - Charlson Comorbidity Index	-0.003	0.044	-0.089	0.084	0.004	0.949
MSI 8H - LODS score	-0.042	0.040	-0.120	0.036	1.104	0.293
MSI 8H - OASIS score	-0.058	0.037	-0.130	0.015	2.423	0.120
MSI 8H - Initial lactate level	-0.052	0.041	-0.132	0.027	1.660	0.198
MSI 8H - Initial arterial PH	0.033	0.042	-0.050	0.115	0.604	0.437
SOFA score day-1 - Charlson Comorbidity Index	0.072	0.049	-0.025	0.168	2.105	0.147
SOFA score day-1 - LODS score	0.033	0.032	-0.031	0.096	1.025	0.311
SOFA score day-1 - OASIS score	0.017	0.029	-0.039	0.073	0.341	0.559
SOFA score day-1 - Initial lactate level	0.022	0.048	-0.073	0.117	0.208	0.648
**SOFA score day-1 - Initial arterial PH**	0.107	0.045	0.019	0.195	5.736	**0.017**
Charlson Comorbidity Index - LODS score	-0.039	0.040	-0.118	0.040	0.933	0.334
Charlson Comorbidity Index - OASIS score	-0.055	0.042	-0.136	0.027	1.744	0.187
Charlson Comorbidity Index - Initial lactate level	-0.050	0.041	-0.130	0.031	1.441	0.230
Charlson Comorbidity Index - Initial arterial PH	0.036	0.044	-0.051	0.122	0.646	0.421
LODS score - OASIS score	-0.016	0.021	-0.056	0.024	0.603	0.437
LODS score - Initial lactate level	-0.011	0.044	-0.097	0.076	0.057	0.811
LODS score - Initial arterial PH	0.074	0.038	0.000	0.149	3.842	0.050
OASIS score - Initial lactate level	0.005	0.040	-0.073	0.084	0.018	0.892
**OASIS score - Initial arterial PH**	0.090	0.036	0.021	0.160	6.430	**0.011**
**Initial lactate level - Initial arterial PH**	0.085	0.034	0.018	0.152	6.254	**0.012**

Delong test (8 hour): hospital mortality

Variables	Estimate	Standard Error	95% CI		Chi-Square	p
			Lower Limit	Upper Limit		
SI 8H - DSI 8H	-0.002	0.009	-0.020	0.016	0.040	0.841
SI 8H - MSI 8H	0.001	0.005	-0.009	0.012	0.060	0.806
SI 8H - SOFA score day-1	0.001	0.017	-0.032	0.034	0.002	0.964
SI 8H - Charlson Comorbidity Index	-0.035	0.022	-0.077	0.008	2.532	0.112
SI 8H - LODS score	-0.037	0.021	-0.079	0.005	3.028	0.082
SI 8H - OASIS score	-0.004	0.018	-0.039	0.031	0.057	0.812
SI 8H - Initial lactate level	-0.023	0.018	-0.059	0.013	1.539	0.215
SI 8H - Initial arterial PH	0.011	0.013	-0.014	0.037	0.722	0.396
DSI 8H - MSI 8H	0.003	0.006	-0.009	0.015	0.282	0.595
DSI 8H - SOFA score day-1	0.003	0.019	-0.034	0.039	0.020	0.887
DSI 8H - Charlson Comorbidity Index	-0.033	0.023	-0.078	0.012	2.034	0.154
DSI 8H - LODS score	-0.035	0.022	-0.078	0.007	2.688	0.101
DSI 8H - OASIS score	-0.002	0.020	-0.041	0.037	0.015	0.904
DSI 8H - Initial lactate level	-0.021	0.020	-0.060	0.019	1.070	0.301
DSI 8H - Initial arterial PH	0.013	0.016	-0.018	0.044	0.656	0.418
MSI 8H - SOFA score day-1	-0.001	0.017	-0.035	0.034	0.001	0.976
MSI 8H - Charlson Comorbidity Index	-0.036	0.022	-0.079	0.007	2.646	0.104
MSI 8H - LODS score	-0.039	0.021	-0.080	0.003	3.290	0.070
MSI 8H - OASIS score	-0.006	0.019	-0.042	0.031	0.089	0.765
MSI 8H - Initial lactate level	-0.024	0.019	-0.060	0.012	1.680	0.195
MSI 8H - Initial arterial PH	0.010	0.014	-0.018	0.037	0.483	0.487
SOFA score day-1 - Charlson Comorbidity Index	-0.035	0.023	-0.080	0.009	2.482	0.115
**SOFA score day-1 - LODS score**	-0.038	0.018	-0.073	-0.004	4.664	**0.031**
SOFA score day-1 - OASIS score	-0.005	0.016	-0.036	0.026	0.105	0.746
SOFA score day-1 - Initial lactate level	-0.024	0.018	-0.058	0.011	1.792	0.181
SOFA score day-1 - Initial arterial PH	0.010	0.013	-0.015	0.036	0.613	0.434
Charlson Comorbidity Index - LODS score	-0.003	0.026	-0.054	0.049	0.010	0.922
Charlson Comorbidity Index - OASIS score	0.030	0.024	-0.017	0.078	1.560	0.212
Charlson Comorbidity Index - Initial lactate level	0.012	0.023	-0.034	0.058	0.262	0.609
**Charlson Comorbidity Index - Initial arterial PH**	0.046	0.021	0.004	0.087	4.713	**0.030**
**LODS score - OASIS score**	0.033	0.015	0.004	0.062	4.976	**0.026**
LODS score - Initial lactate level	0.015	0.024	-0.033	0.062	0.360	0.549
**LODS score - Initial arterial PH**	0.048	0.021	0.007	0.090	5.271	**0.022**
OASIS score - Initial lactate level	-0.018	0.021	-0.060	0.023	0.762	0.383
OASIS score - Initial arterial PH	0.015	0.016	-0.017	0.047	0.882	0.348
**Initial lactate level - Initial arterial PH**	0.034	0.017	0.001	0.066	4.105	**0.043**

Supplementary Figure 5. Receiver operating characteristic (ROC) curves at 8 hour to predict 3-day and in-hospital mortality.

Supplementary Table 5. Cut-off values for SI, DSI, and MSI at 8 hour, Charlson Comorbidity Index, LODS score, OASIS score, and SOFA score, initial lactate level, and initial arterial PH as determined by ROC analysis and Youden's index

	SI	DSI	MSI	Charlson Comorbidity Index	LODS	OASIS	SOFA	Initial lactate level	Initial arterial PH
**3-day mortality**									
**Cut-off**	**0.65**	**1.62**	**1.56**	**7.00**	**9.00**	**46.00**	**5.00**	**7.34**	**1.40**
Spec.	86.1%	65.7%	75.2%	76.4%	74.5%	71.8%	85.5%	62.5%	87.9%
Sens.	54.6%	69.6%	63.6%	52.7%	70.1%	73.1%	66.1%	75.4%	53.8%
PPV	97.7%	95.6%	96.5%	95.9%	96.7%	96.5%	98.8%	95.0%	97.7%
NPV	15.2%	16.9%	16.3%	13.3%	19.2%	20.3%	12.6%	21.2%	16.5%
**In-hospital mortality**									
**Cut-off**	**0.78**	**1.80**	**1.43**	**5.00**	**6.00**	**33.00**	**8.00**	**7.35**	**1.80**
Spec.	68.0%	65.6%	64.9%	60.3%	76.8%	78.8%	69.6%	60.9%	76.4%
Sens.	57.3%	59.6%	59.9%	64.4%	58.2%	53.9%	57.8%	64.2%	54.2%
PPV	85.4%	85.0%	84.8%	84.1%	89.1%	89.2%	87.4%	81.7%	86.3%
NPV	32.8%	33.2%	33.0%	34.2%	36.0%	34.4%	31.2%	38.6%	37.7%

Adjusted for age, gender, race, and ICU care unit.

DSI, Diastolic Shock Index; MSI, Modified Shock Index; NPV, negative predictive value; LODS, Logistic Organ Dysfunction System; OASIS, Oxford Acute Severity of Illness Score; PPV, positive predictive value; ROC, receiver operating characteristic; SI, Shock Index; SOFA, Sequential Organ Failure Assessment; Sens., sensitivity; Spec., specificity; VPs, Start of Vasopressors.

A

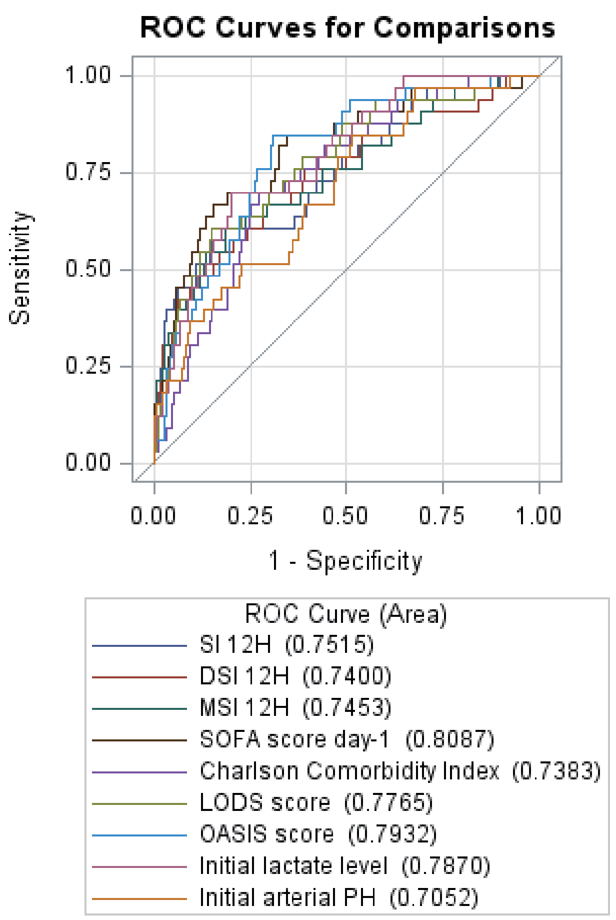

B

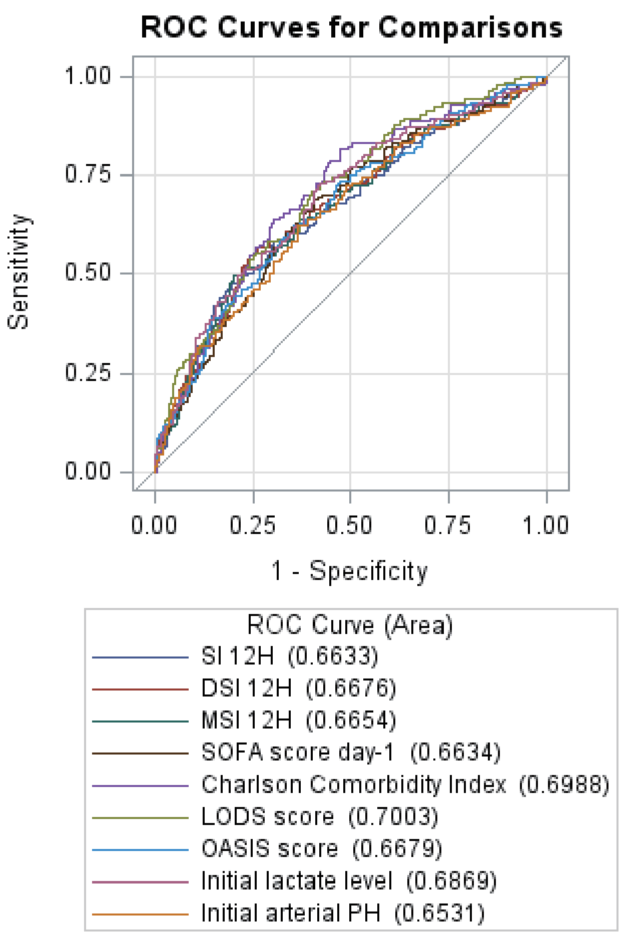

AUC – ROC (12 hour): mortality day-3

Variables	AUC	Error tip.	p	95% CI	
				Lower Limit	Upper Limit
SI 12H	0.752	0.048	<0.0001	0.658	0.846
DSI 12H	0.740	0.050	<0.001	0.642	0.838
MSI 12H	0.745	0.049	<0.0001	0.649	0.842
SOFA score day-1	0.809	0.041	<0.0001	0.728	0.890
Charlson Comorbidity Index	0.738	0.040	0.009	0.660	0.817
LODS score	0.777	0.043	<0.0001	0.692	0.861
OASIS score	0.793	0.037	<0.0001	0.721	0.866
Initial lactate level	0.787	0.038	<0.0001	0.713	0.861
Initial arterial PH	0.705	0.046	0.013	0.616	0.794

AUC – ROC (12 hour): hospital mortality

Variables	AUC	Error tip.	p	95% CI	
				Lower Limit	Upper Limit
SI 12H	0.663	0.027	0.015	0.610	0.717
DSI 12H	0.668	0.027	0.003	0.614	0.721
MSI 12H	0.665	0.027	0.007	0.612	0.718
SOFA score day-1	0.663	0.026	0.009	0.612	0.715
Charlson Comorbidity Index	0.699	0.025	<0.0001	0.649	0.749
LODS score	0.700	0.025	<0.0001	0.651	0.749
OASIS score	0.668	0.027	<0.001	0.616	0.720
Initial lactate level	0.687	0.026	<0.0001	0.636	0.738
Initial arterial PH	0.653	0.027	0.266	0.600	0.706

Delong test (12 hour): mortality day-3

Variables	Estimate	Standard Error	95% CI Lower Limit	95% CI Upper Limit	Chi-Square	p
SI 12H - DSI 12H	0.012	0.019	-0.026	0.050	0.356	0.551
SI 12H - MSI 12H	0.006	0.015	-0.024	0.036	0.169	0.681
SI 12H - SOFA score day-1	-0.057	0.048	-0.151	0.036	1.443	0.230
SI 12H - Charlson Comorbidity Index	0.013	0.041	-0.067	0.093	0.105	0.746
SI 12H - LODS score	-0.025	0.040	-0.103	0.053	0.393	0.531
SI 12H - OASIS score	-0.042	0.037	-0.115	0.031	1.249	0.264
SI 12H - Initial lactate level	-0.036	0.041	-0.115	0.044	0.757	0.384
SI 12H - Initial arterial PH	0.046	0.045	-0.041	0.134	1.079	0.299
DSI 12H - MSI 12H	-0.005	0.012	-0.028	0.018	0.202	0.653
DSI 12H - SOFA score day-1	-0.069	0.047	-0.160	0.023	2.179	0.140
DSI 12H - Charlson Comorbidity Index	0.002	0.040	-0.076	0.080	0.002	0.966
DSI 12H - LODS score	-0.036	0.037	-0.109	0.036	0.981	0.322
DSI 12H - OASIS score	-0.053	0.036	-0.124	0.017	2.182	0.140
DSI 12H - Initial lactate level	-0.047	0.041	-0.127	0.033	1.340	0.247
DSI 12H - Initial arterial PH	0.035	0.036	-0.037	0.106	0.917	0.338
MSI 12H - SOFA score day-1	-0.063	0.047	-0.156	0.029	1.824	0.177
MSI 12H - Charlson Comorbidity Index	0.007	0.043	-0.076	0.090	0.027	0.869
MSI 12H - LODS score	-0.031	0.038	-0.106	0.044	0.664	0.415
MSI 12H - OASIS score	-0.048	0.037	-0.120	0.024	1.706	0.192
MSI 12H - Initial lactate level	-0.042	0.039	-0.117	0.034	1.177	0.278
MSI 12H - Initial arterial PH	0.040	0.040	-0.038	0.118	1.026	0.311
SOFA score day-1 - Charlson Comorbidity Index	0.070	0.047	-0.022	0.163	2.244	0.134
SOFA score day-1 - LODS score	0.032	0.031	-0.029	0.093	1.085	0.298
SOFA score day-1 - OASIS score	0.016	0.028	-0.038	0.070	0.320	0.572
SOFA score day-1 - Initial lactate level	0.022	0.047	-0.070	0.113	0.216	0.642
**SOFA score day-1 - Initial arterial PH**	0.104	0.043	0.018	0.189	5.685	**0.017**
Charlson Comorbidity Index - LODS score	-0.038	0.038	-0.113	0.037	0.993	0.319
Charlson Comorbidity Index - OASIS score	-0.055	0.039	-0.132	0.022	1.949	0.163
Charlson Comorbidity Index - Initial lactate level	-0.049	0.039	-0.126	0.028	1.547	0.214
Charlson Comorbidity Index - Initial arterial PH	0.033	0.042	-0.049	0.115	0.628	0.428
LODS score - OASIS score	-0.017	0.020	-0.055	0.022	0.717	0.397
LODS score - Initial lactate level	-0.011	0.043	-0.095	0.074	0.061	0.805
LODS score - Initial arterial PH	0.071	0.037	-0.001	0.144	3.746	0.053
OASIS score - Initial lactate level	0.006	0.039	-0.070	0.082	0.025	0.874
**OASIS score - Initial arterial PH**	0.088	0.035	0.020	0.156	6.402	**0.011**
**Initial lactate level - Initial arterial PH**	0.082	0.033	0.017	0.146	6.192	**0.013**

Delong test (12 hour): hospital mortality

Variables	Estimate	Standard Error	95% CI Lower Limit	95% CI Upper Limit	Chi-Square	p
SI 12H - DSI 12H	-0.004	0.010	-0.024	0.015	0.198	0.657
SI 12H - MSI 12H	-0.002	0.006	-0.014	0.009	0.134	0.714
SI 12H - SOFA score day-1	0.000	0.017	-0.033	0.033	0.000	0.994
SI 12H - Charlson Comorbidity Index	-0.036	0.022	-0.078	0.007	2.681	0.102
SI 12H - LODS score	-0.037	0.021	-0.079	0.005	2.987	0.084
SI 12H - OASIS score	-0.005	0.018	-0.040	0.030	0.068	0.794
SI 12H - Initial lactate level	-0.024	0.019	-0.060	0.013	1.614	0.204
SI 12H - Initial arterial PH	0.010	0.013	-0.016	0.036	0.574	0.449
DSI 12H - MSI 12H	0.002	0.006	-0.009	0.014	0.143	0.705
DSI 12H - SOFA score day-1	0.004	0.019	-0.033	0.041	0.050	0.823
DSI 12H - Charlson Comorbidity Index	-0.031	0.023	-0.077	0.014	1.826	0.177
DSI 12H - LODS score	-0.033	0.022	-0.075	0.010	2.268	0.132
DSI 12H - OASIS score	0.000	0.020	-0.040	0.039	0.000	0.988
DSI 12H - Initial lactate level	-0.019	0.021	-0.060	0.021	0.874	0.350
DSI 12H - Initial arterial PH	0.015	0.017	-0.018	0.047	0.756	0.385
MSI 12H - SOFA score day-1	0.002	0.018	-0.033	0.037	0.013	0.910
MSI 12H - Charlson Comorbidity Index	-0.033	0.022	-0.077	0.010	2.243	0.134
MSI 12H - LODS score	-0.035	0.021	-0.077	0.007	2.646	0.104
MSI 12H - OASIS score	-0.002	0.019	-0.040	0.035	0.017	0.896
MSI 12H - Initial lactate level	-0.022	0.019	-0.059	0.016	1.257	0.262
MSI 12H - Initial arterial PH	0.012	0.015	-0.017	0.042	0.673	0.412
SOFA score day-1 - Charlson Comorbidity Index	-0.035	0.022	-0.079	0.008	2.604	0.107
**SOFA score day-1 - LODS score**	-0.037	0.017	-0.071	-0.003	4.469	**0.035**
SOFA score day-1 - OASIS score	-0.005	0.015	-0.034	0.025	0.088	0.766
SOFA score day-1 - Initial lactate level	-0.024	0.017	-0.058	0.010	1.842	0.175
SOFA score day-1 - Initial arterial PH	0.010	0.013	-0.015	0.035	0.664	0.415
Charlson Comorbidity Index - LODS score	-0.001	0.026	-0.052	0.049	0.003	0.955
Charlson Comorbidity Index - OASIS score	0.031	0.024	-0.016	0.078	1.682	0.195
Charlson Comorbidity Index - Initial lactate level	0.012	0.023	-0.033	0.057	0.265	0.607
**Charlson Comorbidity Index - Initial arterial PH**	0.046	0.021	0.005	0.086	4.865	**0.027**
**LODS score - OASIS score**	0.032	0.015	0.004	0.061	4.914	**0.027**
LODS score - Initial lactate level	0.013	0.024	-0.034	0.060	0.310	0.578
**LODS score - Initial arterial PH**	0.047	0.021	0.006	0.088	5.154	**0.023**
OASIS score - Initial lactate level	-0.019	0.021	-0.060	0.022	0.826	0.364
OASIS score - Initial arterial PH	0.015	0.016	-0.016	0.046	0.873	0.350
**Initial lactate level - Initial arterial PH**	0.034	0.017	0.001	0.067	4.091	**0.043**

Supplementary Figure 6. Receiver operating characteristic (ROC) curves at 12 hour to predict 3-day and in-hospital mortality.

Supplementary Table 6. Cut-off values for SI, DSI, and MSI at 12 hour, Charlson Comorbidity Index, LODS score, OASIS score, and SOFA score, initial lactate level, and initial arterial PH as determined by ROC analysis and Youden's index

	SI	DSI	MSI	Charlson Comorbidity Index	LODS	OASIS	SOFA	Initial lactate level	Initial arterial PH
**3-day mortality**									
**Cut-off**	**0.96**	**1.70**	**1.13**	**7.00**	**9.00**	**46.00**	**5.00**	**7.34**	**1.40**
Spec.	65.1%	56.0%	75.2%	76.4%	74.5%	71.8%	85.5%	62.5%	87.9%
Sens.	79.3%	82.1%	67.4%	52.7%	70.1%	73.1%	66.1%	75.4%	53.8%
PPV	96.0%	95.2%	96.7%	95.9%	96.7%	96.5%	98.8%	95.0%	97.7%
NPV	23.0%	22.9%	17.9%	13.3%	19.2%	20.3%	12.6%	21.2%	16.5%
**In-hospital mortality**									
**Cut-off**	**1.30**	**1.33**	**1.32**	**5.00**	**6.00**	**33.00**	**8.00**	**7.35**	**1.80**
Spec.	50.0%	74.3%	51.4%	60.3%	76.8%	78.8%	69.6%	60.9%	76.4%
Sens.	75.1%	49.8%	72.5%	64.4%	58.2%	53.9%	57.8%	64.2%	54.2%
PPV	83.0%	86.3%	83.0%	84.1%	89.1%	89.2%	87.4%	81.7%	86.3%
NPV	38.2%	31.3%	36.4%	34.2%	36.0%	34.4%	31.2%	38.6%	37.7%

Adjusted for age, gender, race, and ICU care unit.

DSI, Diastolic Shock Index; MSI, Modified Shock Index; NPV, negative predictive value; LODS, Logistic Organ Dysfunction System; OASIS, Oxford Acute Severity of Illness Score; PPV, positive predictive value; ROC, receiver operating characteristic; SI, Shock Index; SOFA, Sequential Organ Failure Assessment; Sens., sensitivity; Spec., specificity; VPs, Start of Vasopressors.

Supplementary Table 7. Characteristics of 1266 shock patients by quartiles of Modified Shock Index before the start of vasopressor therapy

Covariate	Overall	Quartiles of Pre-VPs MSI				
		Q1	Q2	Q3	Q4	*P* for trend [a]
n	1266	316	317	316	317	
Pre-VPs MSI	1.38 (1.37, 1.40) [b]	0.94 (0.92, 0.95)	1.24 (1.23, 1.25)	1.49 (1.48, 1.50)	1.97 (1.92, 2.02)	
Initial lactate level	3.40 (3.18, 3.63)	3.23 (2.72, 3.73)	3.14 (2.69, 3.58)	3.23 (2.81, 3.66)	3.96 (3.53, 4.40)	0.03
Initial arterial PH	7.31 (7.30, 7.32)	7.32 (7.30, 7.33)	7.31 (7.30, 7.33)	7.31 (7.29, 7.32)	7.29 (7.27, 7.31)	0.09
Initial base excess	-4.49 (-4.94, -4.03)	-3.53 (-4.43, -2.64)	-3.80 (-4.68, -2.92)	-4.64 (-5.51, -3.77)	-5.88 (-6.83, -4.94)	0.001

MSI, Modified Shock Index; Q, Quartile; VPs, Start of Vasopressors.

a Analysis of covariance or logistic regression analysis.

b Continuous variables were presented as mean (95% confidence interval) (all such values).

Supplementary Table 8. Characteristics of 1266 shock patients by quartiles of Diastolic Shock Index before the start of vasopressor therapy

Covariate	Overall	Quartiles of Pre-VPs DSI				
		Q1	Q2	Q3	Q4	*P* for trend [a]
n	1266	316	317	316	317	
Pre-VPs DSI	1.68 (1.66, 1.70) [b]	1.11 (1.09, 1.13)	1.51 (1.50, 1.52)	1.80 (1.79, 1.81)	2.43 (2.35, 2.51)	
Initial lactate level	3.40 (3.18, 3.63)	3.05 (2.55, 3.56)	3.27 (2.79, 3.75)	3.20 (2.80, 3.61)	3.95 (3.53, 4.38)	0.02
Initial arterial PH	7.31 (7.30, 7.32)	7.31 (7.30, 7.33)	7.32 (7.30, 7.33)	7.31 (7.30, 7.33)	7.28 (7.27, 7.30)	0.02
Initial base excess	-4.49 (-4.94, -4.03)	-3.26 (-4.18, -2.34)	-3.87 (-4.78, -2.96)	-4.56 (-5.40, -3.72)	-6.00 (-6.91, -5.08)	<0.001

DSI, Diastolic Shock Index; Q, Quartile; VPs, Start of Vasopressors.

a Analysis of covariance or logistic regression analysis.

b Continuous variables were presented as mean (95% confidence interval) (all such values).
